# Non-viral Gene Delivery Methods for Bone and Joints

**DOI:** 10.3389/fbioe.2020.598466

**Published:** 2020-11-19

**Authors:** Benjamin Gantenbein, Shirley Tang, Julien Guerrero, Natalia Higuita-Castro, Ana I. Salazar-Puerta, Andreas S. Croft, Amiq Gazdhar, Devina Purmessur

**Affiliations:** ^1^Tissue Engineering for Orthopaedics and Mechanobiology, Department for BioMedical Research (DBMR), Faculty of Medicine, University of Bern, Bern, Switzerland; ^2^Department of Orthopaedic Surgery and Traumatology, Inselspital, Bern University Hospital, University of Bern, Bern, Switzerland; ^3^Department of Biomedical Engineering and Department of Orthopaedics, Spine Research Institute Dorothy M. Davis Heart and Lung Research Institute, The Ohio State University, Columbus, OH, United States; ^4^Department of Biomedical Engineering and Department of Surgery, Dorothy M. Davis Heart and Lung Research Institute, The Ohio State University, Columbus, OH, United States; ^5^Department of Pulmonary Medicine, Inselspital, University Hospital, University of Bern, Bern, Switzerland

**Keywords:** non-viral gene delivery, bone, tendon, cartilage, intervertebral disk, GDF5, FOXF1, BMP2

## Abstract

Viral carrier transport efficiency of gene delivery is high, depending on the type of vector. However, viral delivery poses significant safety concerns such as inefficient/unpredictable reprogramming outcomes, genomic integration, as well as unwarranted immune responses and toxicity. Thus, non-viral gene delivery methods are more feasible for translation as these allow safer delivery of genes and can modulate gene expression transiently both *in vivo*, *ex vivo*, and *in vitro*. Based on current studies, the efficiency of these technologies appears to be more limited, but they are appealing for clinical translation. This review presents a summary of recent advancements in orthopedics, where primarily bone and joints from the musculoskeletal apparatus were targeted. In connective tissues, which are known to have a poor healing capacity, and have a relatively low cell-density, i.e., articular cartilage, bone, and the intervertebral disk (IVD) several approaches have recently been undertaken. We provide a brief overview of the existing technologies, using nano-spheres/engineered vesicles, lipofection, and *in vivo* electroporation. Here, delivery for microRNA (miRNA), and silencing RNA (siRNA) and DNA plasmids will be discussed. Recent studies will be summarized that aimed to improve regeneration of these tissues, involving the delivery of bone morphogenic proteins (BMPs), such as BMP2 for improvement of bone healing. For articular cartilage/osteochondral junction, non-viral methods concentrate on targeted delivery to chondrocytes or MSCs for tissue engineering-based approaches. For the IVD, growth factors such as GDF5 or GDF6 or developmental transcription factors such as *Brachyury* or FOXF1 seem to be of high clinical interest. However, the most efficient method of gene transfer is still elusive, as several preclinical studies have reported many different non-viral methods and clinical translation of these techniques still needs to be validated. Here we discuss the non-viral methods applied for bone and joint and propose methods that can be promising in clinical use.

## Introduction

Non-viral gene therapy holds great premises as it is assumed to be less toxic for the host and much safer in terms of gene delivery compared to viral vectors (NIH Report, 2002; Kaiser, 2007).

**FIGURE 1:**
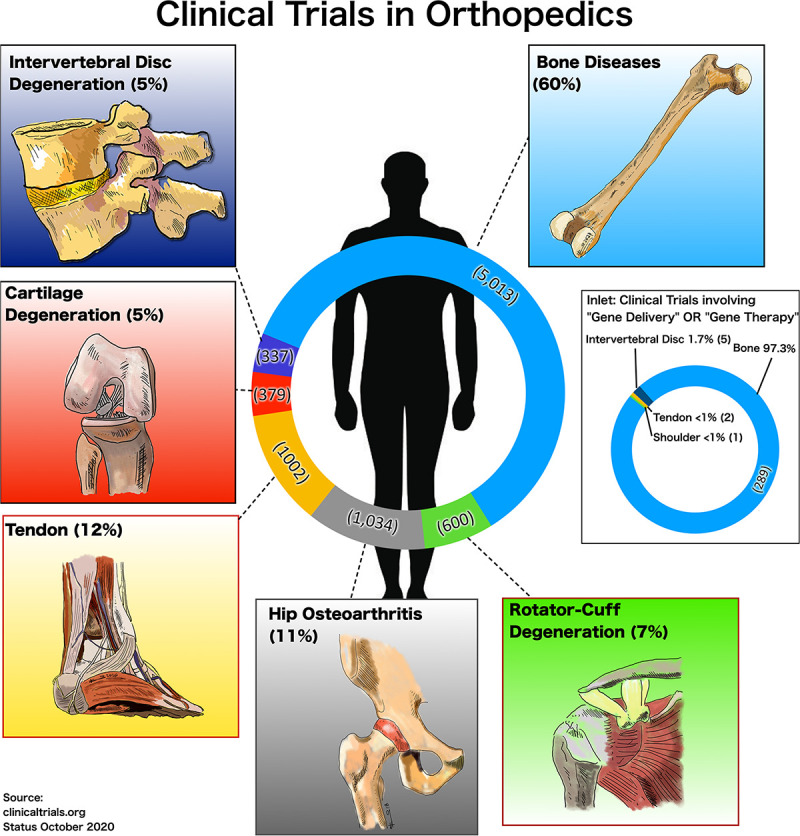
Number and percentages of clinical trials accessed on the 9-October-2020 at ClinicalTrials.gov for different fields in orthopedics, and for inlet limiting the search for the search terms “gene delivery” OR “viral gene therapy” AND the respective area in orthopedics, i.e., “general bone diseases,” “hip,” “tendon,” “cartilage,” and “intervertebral disk degeneration.”

Generally, gene transfer approaches in clinical trials are much less common than clinical trials in general that may involve drug testing (Figure 1). In the clinical trial register (clinicaltrials.gov accessed on 9-October-2020) there were 5,013 (60%) studies reported on “general bone diseases,” 1,034 (11%) on the “hip”-joint, 600 (7%) studies on “rotator cuff,” 1,002 (12%) studies on “tendon” repair, 337 (5%) studies on “intervertebral disk degeneration” (IVD), and 379 (5%) studies on cartilage repair (“cartilage”) (Figure 1). However, with the additional mesh-terms “gene delivery” OR “viral gene therapy” combined with the afore-mentioned orthopedic “specialties” 289 studies were identified for “bone,” only two for the “tendon” and five were found for “IVD” and none for “cartilage” (inlet, Figure 1). Finally, “non-viral” AND “gene delivery” resulted in “zero” studies in all fields of orthopedics. This fact reflects the current situation of non-viral gene delivery trials in this field. One reason might be that the search for new gene therapies, which target certain tissues and cells, has become more cumbersome due to increased levels of regulation (Boissier and Bessis, 1997; Evans et al., 2006, 2012). Many of the recently developed products have not been translated into the clinics, for which many reasons have been identified. One important aspect is safety. The risks and the acceptance of viral gene transfer methods experienced have been affected by sudden patient deaths, such as the examples of Jesse Gelsinger and Joli Mohr (Wilson, 2009; Yarborough and Sharp, 2009). Thus, non-viral gene therapy seems an attractive alternative to viral gene delivery and is an new and emerging field being applied to regenerative medicine. It offers a safer approach to viral vectors with lack of immunogenicity and host genome integration. However, pre-clinical application of such technologies to the musculoskeletal field is still limited.

Many of the alternate approaches are less efficient than viral delivery systems (NIH Report, 2002; Pranatharthiharan et al., 2013; O’Reilly et al., 2015) and due to necessary optimization that is required increases developmental costs exponentially as the product approaches market release (Epstein, 1991; Evans et al., 2012). Another current challenge lies in the experimental designs of clinical trials, which, if not properly planned or randomized, produce doubtful conclusions. As for clinical trials, it needs to be mentioned and clarified if appropriate placebo controls were considered in the original experimental set-up (NIH Report, 2002; Wilson, 2009). In the absence of properly designed controls, it may be impossible to determine whether observed toxicity is due to an underlying disease or the use of a specific vector.

In orthopedic research there are a number of significant health burdens that urgently warrant better therapeutic solutions. In addition to bone metabolic diseases, this also includes problematic musculoskeletal degenerative pathologies of cartilage, tendons, and ligaments, as well as the intervertebral disks (IVDs) of the spine. It has been identified that osteoarthritis (OA) (Wittenauer et al., 2013) and low back pain (LBP) caused by degenerative changes in the IVD are two of the significant global clinical problems to be tackled in the future (GBD 2017 Disease and Injury Incidence and Prevalence Collaborators, 2018). With an increasing elderly population, the demand for joint-replacement surgeries has risen exponentially. For many of the degenerated joints, whether due to aging, genetic predisposition, or trauma, pure mechanical implant solutions exist until now. These do not necessarily take into account the natural tissue properties. Here, in particular in the field of early prevention, non-viral gene therapy could become highly relevant in the near future and is the focus of this review. Here we evaluate promising *in vitro* and *in vivo* non-viral methods being utilized and more specifically in cartilage, the intervertebral disk and bone and gaps/areas that need to be addressed to move these non-viral strategies forward.

## Overview of Non-Viral Vehicle Methods

**FIGURE 2:**
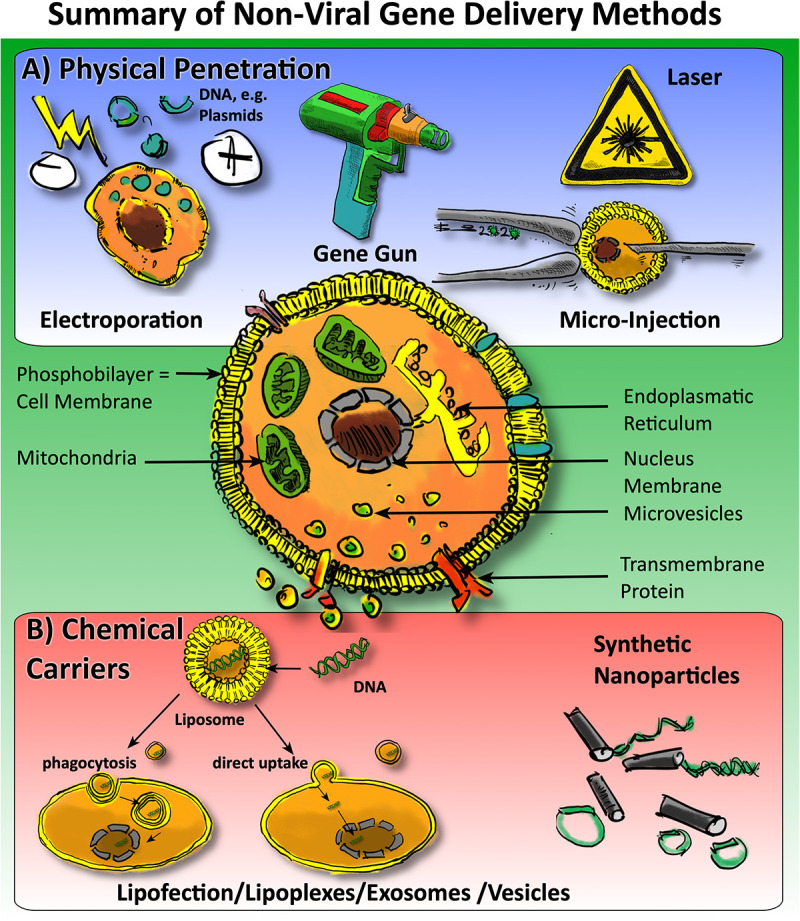
Overview of non-viral approaches for gene delivery to cells in orthopedics. One may generally categorize the methods into **(A)** physical penetration methods (in blue panel) versus **(B)** chemical carriers, i.e., methods involving carriers such as lipofection, micro vesicles, and EVs and, the usage of nanoparticles.

Gene delivery in general may involve the packaging of DNA or RNA in so-called “vectors” but can also be delivered naked (Patil et al., 2019). Generally, one can classify methods according to the approach to overcome the cell’s phosphobilayer membrane: There are “carrier-free” methods that use physical penetration (e.g., electroporation, gene gun, laser, microinjection) or there are methods that use so-called “carriers,” in which DNA or RNA is packed into lipo-philic particles, so-called liposomes, or similar (Figure 2). A distinction can also be made between methods that use fluorescence to monitor the success of the gene transfer or methods that lack this practical feature to monitor the efficiency (Patil et al., 2019). There are several commercial suppliers offering kits that pack DNA or RNA into liposomes and then transfect cells *in vitro* (Figure 2). However, the success of these transfections and duration of the changes may be extremely dependent on the cell-type and the vectors. In some cases, a short over-expression of particular genes is even a warranted side-effect. The advantages of non-viral gene therapy are the fact that the effects are not long-lived. In the following sections, we will briefly introduce the different methods.

### Lipofection/Lipid-Based Vectors

Lipofection via liposomes or lipoplexes has been widely utilized to deliver genetic cargo to cells *in vitro*. This method involves encapsulating pDNA, siRNA, or MicroRNA in spheroids with hydrophilic polar head groups and hydrophobic tails, similar to the structure of the cell membrane (Felgner et al., 1987; Torchilin, 2005). One of the earliest and popular lipofection systems involved cationic lipid, *N*-[1-(2,3-dioleyloxy)propyl]-*N,N*, *N*-trimethylammonium chloride (DOTMA). However, due to initial limitations associated with non-specific protein binding (Lv et al., 2006), more recent iterations have included modifications such as neutral helper lipids to reduce cytotoxicity and to improve the efficiency of the transfection (Gao and Hui, 2001; Dabkowska et al., 2012). These improvements led to the development of lipid nanoparticles, which are formed from cationic lipids with both neutral helper lipids and ionizable cationic amino head groups (Wheeler et al., 1999). These systems can readily form complexes with large nucleic acid constructs and have many advantages such as efficient *in vitro* delivery, biodegradability and the option to tune as well as to functionalize them as described in Table 1. Yet the efficiency of *in vivo* transfection is more limited with these lipid-based methods, as is the optimization of formulations for mass manufacturing. In orthopedics, lipid-based vectors for non-viral gene delivery have been widely utilized (Table 1) such as for treatments for osteoporosis, arthritis, and the IVD.

**TABLE 1:** Advantages and limitations of non-viral gene delivery methods.

Method	Advantages	Limitations	Applications
Electroporation (Gehl, 2003; Wells, 2004; Glover et al., 2005; Mehier-Humbert and Guy, 2005; Liu et al., 2006; Al-Dosari and Gao, 2009; Boukany et al., 2011; Guo and Huang, 2012; Mellott et al., 2013; Wang et al., 2013; Song et al., 2015; Tschon et al., 2016; Tsuchiya et al., 2017; Vroomen et al., 2017; Kawai et al., 2018; Melancon et al., 2018; Shapiro et al., 2018; Shi B. et al., 2018; Tang S. et al., 2019; Bono et al., 2020)	Rapid and straightforward transfection method. Well established protocols for a wide variety of cell lines. High throughput, with the possibility to handle millions of cells per transfection. It can potentially be applied to transfect both dividing and non-dividing cells. Previous studies have reported 100- to 1000-fold increase in gene expression compared to direct injection of naked DNA for transfected tissues (e.g., spinal cord, and cardiac and skeletal muscle).	Transfection efficiency varies significantly depending on the cell line or tissue of origin Limited cell viability due to the high magnitude and non-uniform voltage used (in this method the entire cell surface is exposed to a high intensity electric field), pH changes, and joule heating. High-intensity electric field can lead to DNA instability. Requires direct access/contact with the target tissue, and a large area of tissue for effective transfection. Transfection efficiency can be limited by cargo size. Stochastic transfection profile, where the transgene expression is not homogeneously distributed in the cells/tissue Cargo delivery mechanisms driven in part by endocytosis and endosomal escape, and mediated by binding of the molecular cargo to the cell surface, which may limit transfection efficiency.	Spinal cord and spinal nerves (Tschon et al., 2016) Periodontal tissue (Kawai et al., 2018) Tibial tumor (Melancon et al., 2018) Tumors in liver, lung, and kidney (Vroomen et al., 2017) Bone – Femur (Song et al., 2015) Periodontal ligament (Tsuchiya et al., 2017) IVD (Bucher et al., 2013; May et al., 2017; Tang S. et al., 2019) Skin (Jafari et al., 2018; Pasquet et al., 2018) Lung (Gazdhar et al., 2006; Gazdhar et al., 2007) Heart (Ayuni et al., 2010; Hargrave et al., 2014; Sugrue et al., 2020) Diaphragm (Beshay et al., 2009) Liver (Heller et al., 1996; Kobayashi et al., 2003) Tumor (Goepfert et al., 2011) Cornea (Zhou and Dean, 2007) Retina (Matsuda and Cepko, 2004; Lirong et al., 2014) Brain (Inoue and Krumlauf, 2001; De Fry et al., 2010; Nomura et al., 2016) Artery and muscle (Matsumoto et al., 2001; Zhang et al., 2001; Molnar et al., 2004; Tavakoli et al., 2006; Sokołowska and Błachnio-Zabielska, 2019)
**Nanochannel-based electroporation** (Boukany et al., 2011; Geng and Lu, 2013; Wang and Lee, 2013; Xie et al., 2013; Gao et al., 2014; Chang et al., 2016; Gallego-Perez et al., 2016; Gallego-Perez et al., 2017; Shi B. et al., 2018)	Higher cell viability (approx. 100%), due to selectivity of the cell membrane depending on the size and location of the nanochannels, with <1% of the cell membrane being exposed to the high electric field. High transfection efficiency (81–>95% depending on nano-channel configuration and molecular cargo). Deterministic transfection profile, which leads to high reproducibility. Cargo delivery is solely regulated by electrophoresis forces, which significantly increases the speed of transfection (approximately 3000 times faster compared to bulk electroporation), circumventing endocytosis and endosomal escape.	Requires direct contact with the cell/tissue. Direct delivery is restricted to the outer most cell layer of the tissue.	Induction of pluripotent stem cells (iPSC) (Wang and Lee, 2013; Gao et al., 2014) *In vivo* reprogramming of skin cells into functional induced-neurons and -endothelial cells (Gallego-Perez et al., 2017)
	Dosage control capabilities by adjusting transfection parameters (i.e., voltage, duration, number of pulses). Tissue-nano transfection enables transfection of large sections of tissues *in vivo*. For this method transfection efficacy has been reported to be around 50–250-fold higher compared to standard bulk electroporation (Gallego-Perez et al., 2017).		
**Sonoporation** (Mehier-Humbert and Guy, 2005; Sheyn et al., 2008a; Al-Dosari and Gao, 2009; Wang et al., 2013; Kawai et al., 2018; Bono et al., 2020)	Method that can transfer therapeutic agents into the target cells without surgical intervention (i.e., non-invasive). Enables localized treatment. This method is coupled with real time imaging during the procedure, which allows for closer control of molecular cargo delivery at specific tissue locations.	Low transfection efficiency *in vitro* (approximately 30%). Low reproducibility as its transfection profile is highly stochastic. May cause tissue damage due to overheating conditions. Limited control of energy localization.	Bone engineering (Sheyn et al., 2008a; Feichtinger et al., 2014; Kawai et al., 2018) Tissue regeneration (Nomikou et al., 2016) IVD (Nishida et al., 2006)
**Biolistic gene delivery (Gene gun)** (Kitagawa et al., 2003; Zhu et al., 2004; O’Brien and Lummis, 2006; Al-Dosari and Gao, 2009; Su et al., 2012; Wang et al., 2013; Bono et al., 2020)	Can be applied to a wide variety of cells/tissues Moderate transfection efficiency (around 30–40% *in vitro*) especially for DNA vaccination due to its ability to induce a higher immune response using a lower DNA dose (with up to 100- to 1000-fold increase in antibody production). Ability to transfect non-dividing cells.	Expensive device, reagents, and supplies are required. Can cause significant cell damage due to extensive cell membrane damage. Accumulation of carriers (e.g., gold/tungsten) inside the cells may have a negative impact on cell function. Low reproducibility as its transfection profile is highly stochastic. Transfection efficiency and consistency depend on effective and consistent coating of carriers with the molecular material. Transient transgene expression due to random delivery. Limited tissue depth penetration (less than 1 mm into the skin). Lacks cell specificity.	Immunization (Nomikou et al., 2016) Cancer gene therapy (Kitagawa et al., 2003)
**Engineered extracellular vesicles (microvesicles and exosomes)** (Andaloussi et al., 2013; De Jong et al., 2014; Mulcahy et al., 2014; Lamichhane et al., 2015; Yáñez-Mó et al., 2015; Tkach and Théry, 2016; Maas et al., 2017; Xie et al., 2017; Diomede et al., 2018; Li et al., 2018; van Niel et al., 2018; Marolt Presen et al., 2019; Pizzicannella et al., 2019;	Naturally derived nanocarriers with low immunogenicity. Transfection efficiencies have been reported to vary depending on multiple factors, including size of molecular cargo, extracellular vesicle size and aggregation, and type/origin of recipient cell/tissue. High cargo delivery efficiency. Low cytotoxicity. Can be functionalized for targeted delivery. Innate ability to permeate biological barriers and deliver cargo to target cells.	When directly isolated from non-engineered donor cells may present low reproducibility due to its cargo heterogeneity (influenced by cell type or tissue of origin and isolation methods). Methods to engineer EVs/exosomes post-isolation can be cumbersome and labor-intensive.	Regenerative medicine (Lamichhane et al., 2015) Periodontal ligament (Pizzicannella et al., 2019) Bone tissue repair (Diomede et al., 2018; Li et al., 2018; Chen et al., 2019; Marolt Presen et al., 2019; Trubiani et al., 2019) Joint diseases (OA and periprosthetic infections) (Wu et al., 2019; Rüwald et al., 2020) Soft tissue repair (Mendt et al., 2019) Cartilage regeneration using MSCs (To et al., 2020) Orthopedic tissues (Cappariello et al., 2018)
Pomatto et al., 2019; Trubiani et al., 2019)	High stability in biological fluids and circulation. Size of molecular cargo is not limited by capsid size restrictions. Ability to pack diverse molecular cargo and therapeutic agents.		
**Lipo/polyplex-based carriers** (De Laporte et al., 2006; Basarkar and Singh, 2007; Ditto et al., 2009; Guo and Huang, 2012; Su et al., 2012; Jones et al., 2013; Foldvari et al., 2016; Patil et al., 2019)	Cationic lipoplexes have facilitated cellular uptake due to their positive charge. Can be functionalized with specific ligands to achieve targeted delivery. Moderate transfection efficiency *in vitro* (40–50%). Tunable features (e.g., size, surface properties, molecular cargo) Ability to deliver large DNA constructs.	Cargo delivery relies heavily on endocytosis and endosomal escape. High cytoxicity at higher concentrations (>3:1 lipid: DNA ratio) Low transfection efficiency *in vivo* due to its limited circulation half-life in blood. Non-biodegradable polyplex carriers may accumulate in tissues over time. Cumbersome and labor-intensive production protocols, which limit scalability and reproducibility.	IVD (Kakutani et al., 2006; Chung et al., 2007; Morrey et al., 2008; Sudo and Minami, 2011; Banala et al., 2019) Bone (Winn et al., 2005; Macdonald et al., 2007; Oliveira et al., 2009; Guo-ping et al., 2010; Yan et al., 2014; Monteiro et al., 2014)
**Synthetic polymer-based carriers** (Anderson and Shive, 1997; Prokop et al., 2002; Eliyahu et al., 2005; Pack et al., 2005; De Laporte et al., 2006; Basarkar and Singh, 2007; Ditto et al., 2009; Tzeng et al., 2011; Guo and Huang, 2012; Su et al., 2012; Jones et al., 2013; Foldvari et al., 2016; Patil et al., 2019)	High biocompatibility. Highly effective to prevent molecular cargo degradation and increase stability (e.g., for DNA) Tunable features (e.g., size, surface properties, molecular cargo). Possibility to modulate release rate over time. Ability to be synthesized on a large scale. Transfection efficiencies in the range of 50–75%. Dendrimer configuration enhances gene expression up to 50-fold compared to the bulk polymer.	High cytoxicity at higher concentrations (> 25 kDa) Low transfection efficiency *in vivo* due to its limited circulation half-life in blood (in the range of minutes for some preparations). Small non-degradable polymer carriers may accumulate in tissues over time (e.g., lung and liver). Significant batch to batch variability (e.g., large size distribution, and non-homogenous packing of molecular cargo) depending on fabrication method.	IVD (Feng et al., 2015; Feng et al., 2017) Bone tissue engineering (Dimitriou et al., 2011; Pereira et al., 2020) Bone (Tierney et al., 2012) ch (Itaka et al., 2007; Reckhenrich et al., 2012; Nguyen et al., 2014)
**Natural polymer-based carriers** (Katas and Alpar, 2006; Ji et al., 2009; Yuan et al., 2010; Garcia-Fuentes and Alonso, 2012)	High biocompatibility. Lower cytotoxicity compared to synthetic polymer- and lipid-based carriers. Natural polymers promote more efficient uptake due in part to their ability to cross biological membranes. Transfection efficiency *in vitro* in the range of 30–50% and 50–70% for upregulating or downregulating gene expression, respectively. Ability to be bacteriostatic and anti-inflammatory. Can be used to for applications requiring redosing, as the carrier material will normally degrade in the body.	Ability to enhance tumor accumulation compared to naked siRNA. Low transfection efficiency *in vivo.*	Cancer treatment using chitosan vectors packed with siRNA (Katas and Alpar, 2006) Bone tissue engineering (Bourgeat-Lami, 2002; Kasper et al., 2005, 2006; Stevens et al., 2005; Park et al., 2007; Chew et al., 2011; Wegman et al., 2011, 2014)
**Inorganic-gold nanoparticles** (Olton et al., 2007; Arvizo et al., 2010; Ding et al., 2014; Wegman et al., 2014; Das et al., 2016; Yang et al., 2018)	High biocompatibility. Tunable features (e.g., size and surface coatings). Can be easily functionalized with specific ligands to achieve targeted delivery. Relatively low immunogenicity and cytotoxicity *in vitro.* Transfection efficiency comparable to lipoplexe-based carriers (in the range of 40–55%). Photothermal and other physical properties enable potential implementation in thermal ablation, as contrast agents, or to guide them towards specific tissue niches in the body.	Significant batch to batch variability depending on synthesis technique. Since these carriers present high chemical stability in biological fluids, accumulation inside the cells may have a negative impact on cell function (e.g., cell growth, and tissue viability).	Bone tissue engineering (Olton et al., 2007; Wegman et al., 2014)
**Carbo Nanotubes** (Cai et al., 2005; Liu et al., 2005; Tian et al., 2006; Harrison and Atala, 2007; Moradian et al., 2014; Karimi et al., 2015)	Thermal conductivity. Electrical and mechanical properties. Strength and flexibility. Stability under biological fluids. Ability to sustain release and promote selectivity Can be functionalized to enhance transfection efficiency and targeted delivery. Ability to escape lysosomal pathway. High surface area (∼1300 m^2^/g for closed, single-walled carbon nanotube). Transfection efficiency approximately 4 orders of magnitude higher than for naked DNA.	High fabrication cost. Non-biodegradable. Limited solubility. Low stability under biological fluids, due to possible aggregation. Cytotoxicity and transportation efficiency dependent on their surface functionalization, physical properties, and/or synthesis method.	Tissue engineering (Harrison and Atala, 2007) Drug and gene delivery (Cai et al., 2005; Tian et al., 2006; Moradian et al., 2014; Karimi et al., 2015)

### Electroporation

Electroporation (electro-permeabilization) is a physical method based on the application of high voltage pulses for a short duration to facilitate cellular uptake of nucleic acids or drugs. The concept of electroporation was pioneered by Neumann et al. (1982), and since then it has become a standard method of in vitro transfection due to its low cost and safety (Wong and Neumann, 1982). Optimized electric pulses increase the permeability of the cell membrane through which nucleic acid or drug can enter the cell, once the pulses are terminated the cell membrane rapidly recovers and closes (Gowrishankar et al., 1999; Somiari et al., 2000; Gehl, 2003; Glover et al., 2005; Mehier-Humbert and Guy, 2005; Liu et al., 2006; Al-Dosari and Gao, 2009; Boukany et al., 2011; Guo and Huang, 2012; Mellott et al., 2013; Wang et al., 2013; Song et al., 2015; Tschon et al., 2016; Tsuchiya et al., 2017; Vroomen et al., 2017; Kawai et al., 2018; Melancon et al., 2018; Shapiro et al., 2018; Shi J. et al., 2018; Tang W. et al., 2019; Bono et al., 2020) (Table 1). Over the years, electroporation has also been applied for *in vivo* application, with most applications for preclinical models in skin (Jafari et al., 2018; Pasquet et al., 2018), lung (Gazdhar et al., 2006; Gazdhar et al., 2007) heart (Ayuni et al., 2010; Hargrave et al., 2014; Sugrue et al., 2020) diaphragm (Beshay et al., 2009), liver (Heller et al., 1996; Kobayashi et al., 2003), tumor (Goepfert et al., 2011), cornea (Zhou and Dean, 2007), retina (Matsuda and Cepko, 2004; Lirong et al., 2014), brain (Inoue and Krumlauf, 2001; De Fry et al., 2010; Nomura et al., 2016), artery and muscle (Zhang et al., 2001; Molnar et al., 2004; Tavakoli et al., 2006; Sokołowska and Błachnio-Zabielska, 2019).

*In vivo* electroporation is dependent on various parameters. Therefore, studies have been conducted over to optimize the electrical impulse protocol (voltage, number, and type of pulses), estimation of the interval between the injection of therapeutics and the delivery of electrical pulses, electrode geometry and tissue properties to increase the efficiency of electroporation (Satkauskas et al., 2012; Haberl et al., 2013; Shi B. et al., 2018; Hyder et al., 2020). The mechanism of electroporation mediated nucleic acid and drug delivery is still under investigation. However, detailed research shows that it is a multistep process and involves (i) permeabilization of the plasma membrane under the influence of an electric field, (ii) migration of the DNA/drug toward membrane by electrophoretic forces (iii) and translocation across the membrane. Importantly the mechanisms studied *in vitro* cannot be exactly transferred for *in vivo* electroporation. However, it is agreed that under the influence of an electric filed the cell membrane is being electropermeabilized, which leads to electrophoretically driven migration of nucleic acids and drugs through the target tissue. Therefore, high voltage (HV) and low voltage (LV) pulses have been studied, and their effects have been tested for electropermeabilization.

Various electrodes are used depending on the target site and are of different size shapes and made of different materials. Most commonly, the electrodes are made of stainless steel, copper, titanium, and they differ in their electrical conductivity, price, and corrosion (Rebersek et al., 2014). For the clinical purpose, electrodes made of stainless steel and titanium are used. Recent recommendations suggest using electrodes with a gallium core so that they can absorb the heat generated and thus protect the tissue (Kotnik et al., 2001; Arena et al., 2013). The most commonly used electrodes are either plate electrodes or needle array electrodes. Furthermore, nanochannel-based electroporation has been reported for various applications in Orthopedic research (Boukany et al., 2011; Geng and Lu, 2013; Wang and Lee, 2013; Xie et al., 2013; Gao et al., 2014; Chang et al., 2016; Gallego-Perez et al., 2016, 2017; Shi J. et al., 2018) (Table 1).

### Engineered Vesicles/Exosomes

Extracellular Vesicles (EVs) are cell-derived, lipid membrane enclosed nanoscale particles capable of packaging proteins, lipids, and genetic cargo such as DNA and various RNAs as summarized by O’Brien et al. (2020). They are used for intercellular communication and are excreted by nearly all cells in the body leading to their isolation from most bodily fluids including blood, urine, saliva, amniotic and synovial fluids via ultracentrifugation (Simpson et al., 2008; Andaloussi et al., 2013; Properzi et al., 2013; De Jong et al., 2014; Mulcahy et al., 2014; Lamichhane et al., 2015; Yáñez-Mó et al., 2015; Tkach and Théry, 2016; Maas et al., 2017; Xie et al., 2017; Diomede et al., 2018; Li et al., 2018; van Niel et al., 2018; Marolt Presen et al., 2019; Pizzicannella et al., 2019; Trubiani et al., 2019). Historically, they have been categorized into three main classes mainly based on particle size and biogenesis: Exosomes (40–120 nm) via endolysosomal pathway, Microvesicles/Microparticles (50–1,000 nm) via budding from plasma membrane, and Apoptotic bodies (1–5,000 nm) via blebbing from plasma membrane (Andaloussi et al., 2013; Rilla et al., 2019). However, overlap in the size of these vesicular bodies along with their heterogeneous population, has resulted in interchangeability between the nomenclature (Kowal et al., 2016; Tkach et al., 2017). Thus, micro-vesicles and exosomes will be referred to as EVs in this review.

In general, EVs consist of a lipid bilayer membrane composed of tetraspanins (CD9, CD63, CD81, CD82), integrins, and cell-specific receptors for cell-to-cell communication and internal cargo as described in Wu et al. (2019). Their composition allows for the transmission of proteins, bioactive lipids, and genes, which can alter the function and phenotype of target cells (Andaloussi et al., 2013). Besides, different surface molecules can facilitate ligand-receptor signaling for targeting, adhesion, and fusion to the recipient cell (Boere et al., 2018). Cell-derived EVs can be engineered to carry exogenous genes as a non-viral delivery system as described by Gallego-Perez et al. (2017) via generating EVs from autologous mice fibroblasts and reprogramming them with a cocktail of exogenous of transcription factors into neuronal and endothelial cells. Furthermore, MSC-derived EVs have received growing interest due to their therapeutic potential for joint diseases such as OA and periprosthetic infections, and further characterization of specific therapeutic genetic factors will produce EVs with enhanced regenerative potential (Wu et al., 2019; Rüwald et al., 2020). Thus, these EVs can be engineered both via modification of genetic cargo (electroporation, lipofectamine, siRNA, etc.) or alteration of the EV surface proteins for desired targeting and gene delivery, as summarized in two recent reviews (Sutaria et al., 2017; Mentkowski et al., 2018). EVs offer benefits over conventional delivery systems such as polymers and liposomal systems in terms of stability, immunogenicity, and biocompatibility. Since EVs are generated from innate cells of the body, their size and membrane composition allow for avoidance of degradation *in vivo* through pathways such as lysosomal degradation, endosomal pathway, phagocytosis, or degradation by macrophages as reviewed in Ha et al. (2016). Their small size allows for long term systemic delivery along with the ability to cross the blood-brain barrier and deliver genetic cargo directly into target cell cytosol with high efficiency (Kooijmans et al., 2012; Tran et al., 2015). As EVs are generated from almost all cell types, they are abundant in quantity and can be derived from desired cell types to contain surface markers for cell-specific targeting. They also have advantages over cell therapy due to decreased immunogenicity compared to parent cells because of lower *trans*-membrane MHC proteins and longer shelf life (Ong and Wu, 2015). Despite these advantages, there are some technical and biological challenges still associated with EVs.

Firstly, there are many underexplored areas in EV research, such as their population heterogeneity, differences in isolation methods, and reproducibility, as described in O’Brien et al. (2020). Heterogeneity in EVs can differ between sample to sample as well as within batches due to differences between cell types, culture conditions, and lack of determining specific biomarkers (Nolte-’t Hoen and Wauben, 2012). Isolation methods also vary amongst the field, resulting in heterogeneously isolated EVs with inconsistent naming conventions and make reproducibility difficult (Malda et al., 2016). Besides, their small size also poses disadvantages, as there may be undesired systemic circulation of the generated EV throughout the body. Contradictory findings have also been observed demonstrating the complex nature of EVs such as MSC-derived EVs that both inhibit and promote tumor growth, although EVs themselves do not exhibit the ability to form tumors (Zhu et al., 2012; Bruno et al., 2013).

Current EV related research has primarily focused on MSC derived exosomes, and many are in clinical trials for the treatment and repair of soft tissues (Mendt et al., 2019). Very recently, a systematic review has been conducted on the application of EV to regenerate cartilage using MSCs (To et al., 2020). In these models, all studies that involved MSC-EVs reported less loss of cartilage with the implementation of EVs compared to placebo (To et al., 2020). MicroRNA delivery using EVs has also been a large area of interest. It has shown effects on cell migration, angiogenesis, cell proliferation, and osteogenic differentiation of target cells as summarized in O’Brien et al. (2020). Current research on exosomes/EVs is focused on innate EVs without engineering and their treatment of target cells/tissue. In terms of gene delivery using EVs for orthopedic tissues, this is an unexplored area of research. Thus, EVs demonstrate significant therapeutic potential for non-viral gene delivery due to their intrinsic biocompatibility, low immunogenicity/cytotoxicity, stability, diverse cargo, and engineering capacity. However, there is more elucidation desired before EVs can be used as a gene delivery vehicle in the clinical setting.

### Synthetic Polymer-Based Gene Vectors

Synthetic polymers, both degradable and non-degradable, have several characteristics that make them suitable for gene vector delivery, including biocompatibility, low immunogenicity, high affinity for nucleic acids, improved stability in biological fluids, and the ability to be engineered to mediate cellular entry and endosomal escape (e.g., via hydrophobic modifications) (Anderson and Shive, 1997; Prokop et al., 2002; Pack et al., 2005; Patil et al., 2019). Moreover, their tunable properties and molecular flexibility enable functionalization with specific targeting moieties to favor cell-specific uptake, or conjugation with fusion tags to confirm successful gene delivery (Eliyahu et al., 2005; Guo and Huang, 2012; Foldvari et al., 2016; Patil et al., 2019). Cationic synthetic polymers such as polyamidoamine (PAMAM) dendrimers, polyethyleneimine (PEI), poly[2-(dimethylamino)ethyl methacrylate] (PDMAEMA), poly-Lysine, and polyamidoamine-epichlorohydrin (PAAE), have been widely used for gene delivery applications due to their positive charge, which facilitates genetic cargo loading mediated by their electrostatic interaction with the negatively charged nucleic acids, as well as cellular uptake (De Laporte et al., 2006; Basarkar and Singh, 2007; Patil et al., 2019). Although polycationic-based vectors, such as PEI and PAMAM dendrimers have shown to be effective vehicles for siRNA and miRNA delivery, their highly positive charge may lead to non-specific interactions with the negatively charged phospholipid membrane of circulating cells after systemic delivery (Guo and Huang, 2012; Patil et al., 2019). Cationic polymers can also be modified to modulate their binding strength to the genetic material to achieve successful nucleic acid transfer while still providing viable protection from enzymatic degradation (Jones et al., 2013). The stability of these synthetic polymers can be significantly influenced by their molecular weight, where small complexes with lower molecular weight can be more unstable under physiological conditions, resulting in molecular cargo unpacking, degradation, and clearance (Su et al., 2012). As these small complexes require higher concentrations to achieve adequate gene regulation, if not correctly stabilized, they can aggregate and form larger complexes that can accumulate overtime in organs such as the lung and liver, which significantly impacts cell/tissue function and leads to higher toxicity (Su et al., 2012). A similar phenomenon can be observed for synthetic polymers with higher molecular weight (>25 KDa) (Su et al., 2012). This issue can be addressed by introducing specific surface modifications, such as a PEG-conjugation, to improve steric stabilization and reduce unwanted interactions with salts and other charged or neutral particles present in the circulation (Pack et al., 2005; Su et al., 2012; Jones et al., 2013). Polymers, on the other hand, can help to overcome these limitations by preventing accumulation of the carrier as the genetic material is delivered. For this type of polymer, size and degradation rate can be optimized to favor rapid intracellular delivery (Ditto et al., 2009).

### Advantages and Disadvantages of Current Non-viral Gene Delivery Methods

Viral vectors have developed into the gold standard for modulating gene expression *in vivo* thanks to their high transfection efficiency and ability to bypass endocytosis to enter the cytosol, especially when compared to synthetic transfection methods such as lipo/polyplex-based carriers (Ziello et al., 2010). However, although promising to obtained stable (when using adeno-associated viruses) or transient (when using adenoviruses) transfection of cells, viral vectors present significant limitations due to the persistent risk of triggering immune reactions which hinders the ability for redosing, limited size of the molecular cargo due to capsid size restrictions, and potential biosafety concerns for clinical applications (Daya and Berns, 2008; Joshi et al., 2017). To overcome these limitations many non-viral physical and chemical/biological transfection methods have been developed (e.g., electroporation-based approaches, synthetic nanocarriers, and electro exosomes/EVs) (Wu et al., 2013). However, some of these methods are still limited for example by low stability in biological fluids for synthetic nanocarriers, low transfection efficiency and electro-toxicity for some electroporation-based methods such as bulk electroporation since the entire cell surface is exposed to a high-intensity electric field, and nanocarrier (i.e., gold/tungsten) toxicity for biolistic transfection methods (Al-Dosari and Gao, 2009; Boukany et al., 2011; Wang and Lee, 2013). Nanochannel-based electroporation approaches have emerged as a potent tool to circumvent these limitations. In this type of technology, nanochannel membranes are used to focus a high-intensity electric field applied to the cell membrane, where only the cells in contact with the nanochannels are porated, and the electric field is only applied to a very small portion of the cell membrane equivalent to the area of the nanochannel. This feature improves cell viability and leads to a larger transmembrane potential with enhanced transfection efficiencies and closer control over molecular cargo transfer with a highly deterministic transfection profile, compared to the stochastic profile observed when using bulk electroporation (Boukany et al., 2011; Gallego-Perez et al., 2016). More recently, Gallego-Perez et al. (2017) have used the same governing physical principles to enable transfection of tissues *in vivo* via Tissue Nano-Transfection to induce direct cell reprogramming for regenerative applications (Gallego-Perez et al., 2017). Table 1 provides an overview of advantages and limitations for several widely used non-viral gene delivery techniques, such as electroporation (Gehl, 2003; Wells, 2004; Glover et al., 2005; Lin et al., 2005; Liu et al., 2005, 2006; Mehier-Humbert and Guy, 2005; O’Brien and Lummis, 2006; Al-Dosari and Gao, 2009; Boukany et al., 2011; Tzeng et al., 2011; Guo and Huang, 2012; Mellott et al., 2013; Wang et al., 2013; Ding et al., 2014; Song et al., 2015; Das et al., 2016; Tschon et al., 2016; Tsuchiya et al., 2017; Vroomen et al., 2017; Kawai et al., 2018; Melancon et al., 2018; Shapiro et al., 2018; Shi J. et al., 2018; Pomatto et al., 2019; Tang S. et al., 2019; Bono et al., 2020), Nanochannel-based electroporation (Boukany et al., 2011; Geng and Lu, 2013; Wang and Lee, 2013; Xie et al., 2013; Gao et al., 2014; Gallego-Perez et al., 2016; Chang et al., 2016; Gallego-Perez et al., 2017; Shi J. et al., 2018), Sonoporation (Mehier-Humbert and Guy, 2005; Sheyn et al., 2008a; Al-Dosari and Gao, 2009; Wang et al., 2013; Balmayor and van Griensven, 2015; Kawai et al., 2018; Bono et al., 2020), Biolistic gene delivery (Gene gun) (Kitagawa et al., 2003; Zhu et al., 2004; Al-Dosari and Gao, 2009; Su et al., 2012; Wang et al., 2013; Bono et al., 2020), engineered EVs (microvesicles and exosomes) (Andaloussi et al., 2013; De Jong et al., 2014; Mulcahy et al., 2014; Lamichhane et al., 2015; Yáñez-Mó et al., 2015; Tkach and Théry, 2016; Maas et al., 2017; Xie et al., 2017; Diomede et al., 2018; Li et al., 2018; van Niel et al., 2018; Marolt Presen et al., 2019; Pizzicannella et al., 2019; Trubiani et al., 2019).

## Non-Viral Gene Delivery to Articular Cartilage

Articular cartilage degeneration is a severe pathology and affects about three out of 10 people worldwide (Evans and Robbins, 1999; Wittenauer et al., 2013). There is an increase in interest to deliver gene therapy to the cartilage to rescue or activate remaining chondrocytes or to drive MSCs toward chondrocytes (Evans and Robbins, 1999; Huizinga, 1999; Burstein, 2001; Im, 2016). The clinical problem is that hyaline cartilage cannot be easily regrown *ex vivo*, although the chondrocytes can be expanded after isolation. However, the quality of the matrix that these cells produce differs from native tissue and with inferior biomechanical properties (Gelse et al., 2003). Most methods that have been proposed so far involve the removal of chondrocytes and the *ex vivo* cell expansion, and then in a second step, the cells will be treated with non-viral gene delivery approaches, such as TGFβ, other Bone Morphogenic Proteins (BMPs), or other anabolic genes such as insulin-like growth factor-1 (IGF-1) (Saraf and Mikos, 2006). It has also been shown that autologous chondrocytes seem challenging for successful transfections and other cell sources as adipose or bone-marrow-derived MSCs may be more promising (Heyde et al., 2007). Addressing anti-inflammatory pathways by incorporation of IL-10 or similar cytokines has been tested with promising results (Khoury et al., 2006).

### Lipid-Based Gene Vectors for Cartilage Repair

Lipids were successfully used in a three-step method to achieve high efficiency of transfection by combining permeabilization of primary cells with a mild detergent, by association of pDNA with a polycationic (poly-L-lysine) core covalently linked to a receptor-ligand (transferrin) and addition of cationic liposomes (Goomer et al., 2001). Transfection efficiencies using lipofection reached 40% after 36 h (Stöve et al., 2002). Gene delivery for tissue, which is rich in GAGs, collagens, and other extracellular matrices (ECM) components seems particularly challenging for *in vivo* delivery of DNA. Noteworthy, non-viral gene delivery with FITC-labeled chondrocyte-affinity peptide (CAP) conjugated PEI/DNA particles was investigated in a rabbit knee joint OA-model (Pi et al., 2011). These authors found that by using the CAP-motive that the integration of the PEI/DNA was much more efficient than with placebo. Many more studies were undertaken based on *in vitro* primary cultures (Odabas et al., 2013; Raftery et al., 2016) using rabbit or bovine-derived chondrocytes or even patient-derived chondrocytes. Recently, chondrogenic differentiation was induced from induced pluripotent stem cells (iPSC) using non-viral mini-circle vectors (Rim et al., 2020). The various approaches for cartilage repair to treat rhematoid arthritis (RA) were recently summarized by Pirmardvand Chegini et al. (2018). Here, mainly anti-inflammatory genes like IL-1, IL-6, and IL-10 were influenced by vector transfer. A prominent inducer for the regeneration of cartilage, i.e., SOX9, delivered in non-viral approach has been shown as a promising strategy (Song and Park, 2020). Also, here a wide range of studies used liposome-based methods to transfect primary chondrocytes and MSCs (Goomer et al., 2001; Stöve et al., 2002; Sun et al., 2009).

### Synthetic Polymer-Based Gene Vectors for Cartilage Repair

Recently, Gonzalez-Fernandez et al. (2017) found that if MSCs were transfected with different gene carriers that the morphology of MSCs was highly influenced by the application of different categories of vectors. Generally, studies tried to modulate and activate gene expression of differentiated chondrocytes and/or MSCs. Target genes of interest were SOX9 and collagen type X among others. It was shown that gold-nanoparticles were found to be very efficient to transfer genes to cartilage (Pirmardvand Chegini et al., 2018).

### Physical Gene Vector Methods for Cartilage Repair

Nucleofection through electroporation (EP) has been applied successfully on primary chondrocytes in a high throughput format (Haag et al., 2009). Earlier electroporation has been evaluated in cartilage by Mir et al. (2005) among other tissues to test regenerative effects in cartilage. A more systematic comparison to address whether local administration versus systemic gene electrotransfer (ET) could be more successful would be to apply anti-inflammatory plasmids (Khoury et al., 2006). They found in a mouse OA-model that intra-muscular application of ET was more efficient than intra-articular ET, which is unexpected, given the local administration of the vector to the site of action.

### Exosomes/Extracellular Vesicles for Cartilage Repair

Extracellular vesicles were used successfully to thrive differentiation of MSCs toward chondrocytes *in vitro* and *in vivo* (see also chapter on EVs) (To et al., 2020). It has been shown that cell-derived EVs are involved in the pathogenesis of OA, playing important roles in antigen presentation, inflammation, angiogenesis, cell–cell signal communication, thrombosis, and articular cartilage ECM degradation (Fu et al., 2018; Rilla et al., 2019). It could be shown that even up-regulation of autophagy is involved in the release of EVs in bovine and human degenerated chondrocytes (Rosenthal et al., 2015). It also has been shown that their specific interactions exist between the ECM proteins of articular cartilage and matrix EV’s proteins (Wu et al., 1992). In chondrocytes (but also for osteoblasts and tenocytes) EVs play a key role in the induction of matrix mineralization, these are called matrix vesicles (MVs) (Anderson, 2003). Thus, MVs are involved in the onset of calcification in painful OA-joints (Jubeck et al., 2008). Chondrocytes have been proven *in vitro* to transfer EVs to MSCs in co-culture (Kim et al., 2019). On the other hand, EVs from MSCs activate chondrocytes and lead to an improved ECM (Kim et al., 2019). It was further shown experimentally that cellular proximity was needed to induce EV-associated regenerative effects. Thus, EVs seem to be the perfect vehicle to transfer DNA or RNA as these have been proven to exist naturally, and some do even contain miRNA (Lin et al., 2018).

### Summary of Non-viral Gene Delivery for Cartilage Repair

To summarize, there were many studies conducted in the area of cartilage repair (∼700 in PubMed, starting from 1986 to the present). Unsolved issues concern how EVs interact with components of the ECM of cartilage. It seems clear that hyaluronic acid (HA) and GAGs, such as chondroitin sulfate, are involved in the regulation of EVs and MVs activity (Rilla et al., 2019). Of great interest in the field of cartilage repair is the ability of EVs to transfer bioactive cargo between cells and influence phenotype and behavior directly upon uptake (Gerlach and Griffin, 2016). The EV-mediated delivery of active contents, including cytoplasmic and membrane proteins as well as nucleic acids, and in particular miRNA sequences, has been demonstrated (Gerlach and Griffin, 2016; Rilla et al., 2019). Of specific interest is HA, which interacts via CD44 receptor, and thus could be used as a potential non-viral gene delivery system for chondrocytes. *In vivo*, particular challenges persist in overcoming the barriers of GAG and other ECM components to reach the chondrocytes with EVs or other non-viral vectors.

## Non-Viral Gene Delivery to the Intervertebral Disk

The IVD is the largest avascular and aneural organ in the human body. It is a joint between adjacent vertebrae in the spinal column and facilitates flexion, extension, and rotation of the spine while relying on the diffusion of nutrients through the cartilage endplate of the vertebral body (Urban et al., 2004; Heuer et al., 2008). As a consequence of the avascular nature of this tissue, the healthy mature disk is relatively acellular; few cells existing within a dense ECM of proteoglycans and collagen (Humzah and Soames, 1988). During aging and degeneration, there is a decline in matrix biosynthesis and cellularity, together with an increase in catabolism and inflammation resulting in a loss of IVD structure/function (Antoniou et al., 1996; Le Maitre et al., 2007). These changes create a hostile microenvironment for regenerative strategies that focus on restoring structure and function to the joint while reducing the underlying mechanisms of disease. This, together with logistical and regulatory challenges, pose significant barriers to the success of therapeutic strategies for the IVD, specifically: (i) the lack of continuous drug delivery systems, (ii) reduced sustained cell viability in the hostile microenvironment of the IVD or (iii) regulatory and safety hurdles in the case of viral gene editing that permanently integrates with host DNA which may cause off-target mutations. Current biological strategies for disk repair to date have focused on growth factors, anti-inflammatory drugs, stem cell therapy (adult mesenchymal and iPSC) and viral gene delivery (Sakai et al., 2006; Orozco et al., 2011; Gorth et al., 2014; Hodgkinson et al., 2019) with limited long-term efficacy and safety due to many of the barriers stated above. Non-viral gene delivery strategies for treating the degenerate and painful IVD are receiving increasing attention given their potential for sustained effects on the innate IVD cell phenotype of interest *in situ*; however, this is still an emerging field with relevant studies discussed below, described in Table 2 and categorized based on their mode of delivery.

**TABLE 2:** Summary of non-viral gene delivery for the intervertebral disk.

Chemical Vector/System	Scaffold/matrice or add-on	Wound type	Animals/Cells	Growth Factor or Gene	DNA/RNA	Results	References

*Lipid-based transfection/Lipid-based gene vectors*							
LTI and other Lipid based non-viral reagents	N.D.	*In vitro*	Human IVD Cells	Luciferase	pDNA	LT1 found to be lease toxic our of other lipid based agents, but significantly less efficient compared to Adeno = viral controls. Addition of Hyaluronidase may increase transfection efficiency.	Morrey et al., 2008
Lipofectamine 2000	N.D.	*In vitro*	Human and Rat Nucleus Pulposus Cells	Firefly Luciferase	pDNA and siRNA	Reduction of Firefly luciferase in both rat and human nucleus pulposus cells for two weeks but the disappearance of inhibitory effects by three weeks and a significant decrease in cellular proliferation compared to fibroblast controls.	Kakutani et al., 2006
Lipofectamine and Invivofectamine	N.D.	*In vitro and in vivo*	Rabbit Nucleus Pulposus Cells and annular puncture model	Caspase 3	siRNA	Decreased cell apoptosis in vitro with suppression of degeneration in vivo.	Sudo and Minami, 2011
Lipofectamine	N.D.	*In vitro*	Ovine Nucleus Pulposus Cells	hTERT	pDNA	Increased telomerase activity, cellular lifespan, and collagen I and II Production. However, karyotypic instability warrants method safety.	Chung et al., 2007
Liposomes	N.D.	*In vivo*	Rabbit IVD Puncture	ADAMTS5 and Caspase 3	siRNA	Caspase 3 siRNA and in synergy with ADAMTS5 siRNA limited disk degeneration. However, ADAMTS5 siRNA alone was ineffective in suppressing ADAMTS5. expression	Banala et al., 2019

***Synthetic polymer-based transfections/Synthetic polymer-based gene vectors***							

Mixed polyplex micelles	PEG-poly(N-isopropyl acrylamide Mixture	*In vitro and in vivo*	Rabbit Nucleus pulposus cells and Rat Tail degeneration Model	OH-1	pDNA	High nuclease activity resistance, protein absorption, and increase gene transfection efficiency compared to single bock polymer *in vitro*. OH-1 delivery decreased MMP3 and COX-2 expression *in vitro* with an effective decrease in inflammation and GAG restoration *in vivo* compared to unique block polymer.	Feng et al., 2015
Nano polyplexes	Polyplexes encapsulated in nano-spheres	*In vivo*	Rat Tail degeneration Model	NR4A1	pDNA	Successful delivery of NR4A1 along with limiting fibrosis.	Feng et al., 2017
Injectable MMP degradable hydrogel	MMP responsive polyplex micelles	*In vitro* and *in vivo*	Rabbit Nucleus pulposus cells and Intervertebral Disc Puncture	miRNA-29	miRNA	MMP-responsive polyplex micelles increased the efficiency of cellular uptake and endosomal escape. Limited fibrosus and reduce disc degeneration in rabbit model.	Feng et al., 2018

***Physical transfection methods/Physical gene vectors methods***							

Nucleofector System Bulk Electroporation	PEG Hydrogel suspension for organ culture	*In vitro*	Human MSCs and Bovine papain digest IVD organ culture	GDF5	pDNA	GDF5 expressed in monolayer cell culture up to three weeks up-regulated ACAN, SOX9, KRT19 in transfected cells in a 3D alginate culture. Partial GAG/DNA recovery at 7 days in organ culture.	Bucher et al., 2013
Neon Transfection System Bulk Electroporation	N.D.	*In vitro*	Bovine and human IVD Cells	pCMV6	pDNA	Determined optimal electroporation parameters for delivery into human and bovine IVD cells to be two pulses at 1400 Volts for 20 ms.	May et al., 2017
Neon Transfection System Bulk Electroporation	N.D.	*In vitro*	Human Nucleus Pulposus cells	*Brachyury*	pDNA	Significant increase in *Brachyury*, phenotypic markers, decreased inflammatory/catabolic/pain markers, and increased GAG accumulation over four weeks.	Tang W. et al., 2019
Microbubble-Enhanced Ultrasound	N.D.	*In vivo*	Rat Tail IVD	GFP and Firefly Luciferase	pDNA	Ultrasound transfection significantly enhanced pDNA transfection efficiency into nucleus pulposus cells *in vivo*—transgene expression up to 24 weeks in IVD but declined with time.	Nishida et al., 2006

***Exosomes/Extracellular Vesicles***							

MSC derived exosomes	N.D.	*In vitro* and *in vivo*	Human Nucleus pulposus cells and rattail IVD model	miRNA-21	miRNA	MSC derived exosomes inhibited apoptotic processes PTEN restraints in cells and alleviates nucleus pulposus apoptosis and IVD degeneration *in vivo*.	Cheng et al., 2018

*N.D., non determined.*

### Lipid-Based Gene Vectors for the Intervertebral Disk

Lipid-based gene delivery systems were amongst the first non-viral methods used to investigate the effects of gene transfection on IVD cells. Morrey et al. (2008) screened several lipid-based non-viral agents for gene delivery in human degenerative IVD cells *in vitro* focusing on efficiency, safety, and optimal dose. Out of the seventeen agents assessed, they identified “LT1” as the most efficient and least toxic when compared to other lipid-based agents. When culture medium without antibiotics, buffers, and amino acids was used, including hyaluronidase pre- and post-transfection, these changes to the transfection protocol increased efficiency while maintaining viability. Yet, when compared to the adenoviral associated gene delivery controls, LT1 transfection was significantly less efficient than viral delivery, warranting a need for further optimization of these transfection methods.

Lipofectamine has been used to transfect nucleus pulposus cells with either DNA plasmid vectors as well as small interfering RNAs (siRNAs) *in vitro*. To determine the potential of siRNAs to knockdown gene expression in nucleus pulposus cells isolated from rats and human patients with scoliosis, these cells were co-transfected with reporter luciferase plasmid Firefly and its corresponding siRNA using lipofectamine (Kakutani et al., 2006). The expression of Firefly luciferase was reduced by 94.7 and 93.7% in rat and human nucleus pulposus cells respectively. This demonstrates successful knockdown of “Firefly luciferase” that was maintained for 2 weeks, however, significant decreases in nucleus pulposus cell proliferation were observed compared to the fibroblast control and inhibitory effects of knockdown disappeared by 3 weeks. To investigate the effect of siRNAs on silencing a relevant target associated with disk degeneration, Sudo and Minami (2011) transfected rabbit nucleus pulposus cells with Caspase 3 siRNA *in vitro* and *in vivo* using lipofectamine or “invivofectamine” reagent complex, respectively. Significant decreases in apoptosis *in vitro* and suppression of degenerative changes as observed on MRI and histologically were noted *in vivo* with non-viral delivery of Caspase 3 siRNA. In addition to the non-viral delivery of siRNAs, lipofectamine has been used *in vitro* to transfect ovine nucleus pulposus cells with a plasmid vector containing human telomerase reverse transcriptase (hTERT) to examine effects on cellular lifespan (Chung et al., 2007). hTERT significantly increased telomerase activity, lifespan, and collagen I and II expression relative to vector controls, however, karyotype instability suggested further studies are necessary to validate the safety of this strategy.

Lipid-based vectors such as liposomes have been used to transfect multiple siRNAs into cells *in vitro* and *in vivo*. Transfection of liposomal siRNA for Caspase 3 and A Disintegrin and Metalloproteinase with Thrombospondin motifs-5 (ADAMTS5) was first optimized in a human hepatocellular carcinoma cell line *in vitro* followed by injection of Caspase 3 and ADAMTS5 siRNA alone or in synergy into a rabbit IVD puncture model (Banala et al., 2019). The liposomal siRNA formulations for Caspase 3, including the combined synergy groups, were able to limit IVD degeneration *in vivo* as demonstrated by MRI and histopathology with the limited effect of ADAMTS5 siRNA treatment alone suggesting that the ADAMTS5 siRNA was ineffective at suppressing ADAMTS5 expression. The studies described above highlight the potential of lipid-based transfection and vector-based systems to deliver genes and gene targets successfully to IVD cells *in vitro* and *in vivo*, however, given the limitations associated with transfection efficiency and the few gene targets that have been assessed so far, warrants further optimization of these methods with a diverse array of gene candidates.

### Synthetic Polymer-Based Gene Vectors for the Intervertebral Disk

Synthetic polymer-based gene vectors are attractive alternatives for non-viral gene delivery when compared to viral vectors as they demonstrate low immunogenicity, have tunable structural and surface components, and can be synthesized on a large scale at relatively low-cost (Pack et al., 2005). A limited number of studies have explored the potential of such polymer-based non-viral gene delivery systems to treat IVD cells *in vitro* and *in vivo*. Feng et al. (2015) developed an elegant system to therapeutically deliver pDNA by combining cationic block polymers polyethyleneglycol (PEG)-block-poly (*N*-[*N*-(2-aminoethyl)-2-aminoehtyl]aspartamide) [PEG-b-PAsp(DET)] and poly(*N*-isopropylacrylamide)-block-PAsp(DET) [PNIPAM-b-PAsp(DET)], which they termed “mixed polyplex micelles” (MPMs). These MPMs demonstrated high resistance to nuclease activity and protein absorption including significantly higher gene transfection efficiency in nucleus pulposus cells when compared with single block polymers [PEG-b-PAsp(DET)] *in vitro* and *in vivo*. Furthermore, when MPMs were loaded with heme oxygenase-1 (HO-1), an anti-oxidant and anti-inflammatory, and used to treat nucleus pulposus cells previously stimulated with IL-1β *in vitro*, decreases in matrix metalloproteinase 3 (MMP3) and cyclo-oxygenase-2 (COX-2) were observed. These effects were reproduced in an IVD degeneration rat tail model where MPMs loaded with HO-1 were more effective an decreasing the inflammatory response and restoring glycosaminoglycans (GAG) when compared to the single block polymer loaded vectors. The authors of this study went on to develop new synthetic polymer-based non-viral gene delivery systems for treating IVD degeneration. One involved nano-sized polyplexes that self-assemble into a double-shell structure, which are then encapsulated in biodegradable nano-spheres and co-injected with nanofibrous spongy microspheres, providing a two-stage delivery system with both temporal control and highly efficient delivery of pDNA (Feng et al., 2017). This system was used to successfully deliver the gene encoding anti-fibrotic agent, orphan nuclear receptor 4A1 (NR4A1) to the IVD *in vivo*, and limit fibrosis in a rat tail model of disk degeneration. In a more recent study, Feng et al. (2018) developed an injectable MMP-degradable hydrogel encapsulating MMP-responsive polyplex micelles for continuous and bioresponsive delivery microRNA-29 to limit fibrosis and reduce degeneration in an *in vivo* rabbit puncture model of IVD degeneration. These polyplex non-viral systems described above highlight the potential of synthetic polymers to successfully deliver genes of interest to the degenerate IVD using a variety of small animal models (rat and rabbit) and gene targets with high efficiency and low cytotoxicity. The next steps could include longer-term studies (>12 months) and scaling-up to relevant larger animal models of IVD degeneration such as the sheep, goat, or dog.

### Physical Gene Vector Methods for the Intervertebral Disk

Physical methods for non-viral gene delivery offer a safe and feasible way for transfecting large quantities of cells *in vitro*. Studies by Bucher et al. used Nucleofector technology to electroporate human MSCs with growth factor differentiation factor 5 (GDF5) to transplant these cells in a degenerate bovine IVD organ culture model (Bucher et al., 2013). Monolayer cultures of transfected MSCs expressed GDF5 for up to 3 weeks. When GDF5 transfected MSCs were seeded in alginate beads, key IVD markers ACAN, SOX9, and KRT19 were up-regulated in these cells compared to untransfected cells. When GDF5 transfected MSCs were injected within a PEG hydrogel suspension into the bovine IVD organ culture papain degeneration model, a partial recovery of GAG/DNA was observed after seven days. In a more recent study, May et al. (2017) have used the Neon transfection system to validate parameters of voltage, number and duration of pulses for electroporation mediated gene transfer in bovine and human IVD cells. They determined successful transfection (≥47% efficiency) of commercially available plasmid pCMV6-AC-GFP by flow cytometry with a protocol of two pulses of 1400V for 20ms in bovine and human nucleus pulposus and annulus fibrosus cells. The effect of transfecting *GDF6* was examined using this protocol and system; however, due to potential limitations with the specific *GDF6* plasmid used, no increase in ECM proteins could be observed. Tang et al. used this same Neon transfection system to examine the effect of electroporating developmental transcription factor *Brachyury* into human nucleus pulposus cells from cadavers and patients undergoing surgery for low back pain in 3D *in vitro* culture (Tang S. et al., 2019). In this study, significant increases in *Brachyury* were observed up to 4 weeks, together with improvements in IVD phenotypic markers FOXF1, KRT19, and SOX9 and decreases in inflammatory/catabolic/pain markers IL1−β, IL-6, NGF, and MMP-13 compared to transfected sham vector control cells. Besides, significant increases in glycosaminoglycan accumulation were observed, suggesting that *Brachyury* was able to reprogram degenerate nucleus pulposus cells to a healthier pro-anabolic phenotype, however, since some effects appeared transient, further optimization of the protocol was deemed necessary.

The studies described above highlight the potential and feasibility of using bulk electroporation to deliver genes to IVD cells non-virally. An alternative physical method that has been investigated is Microbubble-Enhanced Ultrasound Gene Therapy. GFP and firefly luciferase reporter plasmids were mixed with microbubbles of ultrasonography contrast agent and injected into the IVDs of rat tails *in vivo* (Nishida et al., 2006). Therapeutic ultrasound was applied to the surface of inserted disks, and the IVD was isolated at 1, 3, 6, 12, and 24 weeks post-injection. Transgene expression was observed up to 24 weeks in the IVD however, overall declined with time suggesting that, while a potentially promising method, further validation of this technique may be necessary.

Physical non-viral transfection of pDNA is an attractive method for delivering genes of interest to the IVD. Electroporation shows promise for *in vitro* gene delivery, however, a direct translation of this method for use *in vivo* in relevant animal models of disk degeneration is more challenging, and this is where other physical techniques such as ultrasound could be used.

### Exosomes/Extracellular Vesicles for the Intervertebral Disk

The therapeutic potential of exosomes and EVs is a new and emerging field. With respect to the IVD, exosomes derived from both human MSCs and nucleus pulposus cells have been shown to promote ECM biosynthesis and enhance IVD phenotypic markers when co-cultured with either nucleus pulposus cells or MSCs, respectively (Lu et al., 2017). A recent study by Cheng et al. (2018) has demonstrated the potential of MSC derived exosomes to deliver specific endogenous cargo in the form of microRNAs to nucleus pulposus cells *in vitro* and *in vivo* suggesting that exosomes could be engineered to deliver specific exogenous pDNA to IVD cells as a method of non-viral gene delivery.

### Summary of Non-viral Delivery Systems for the Intervertebral Disk

Identifying non-viral gene delivery systems for the treatment of IVD degeneration is a research priority given the potential of gene therapy-based approaches to regenerate the IVD using discogenic growth factors, RNA interference/silencing and transcription factors and limitations associated with the use of viral vectors. While this is still a growing area for the IVD, the studies described above highlight the clinical applicability and relevance of these methods as safe and efficacious alternatives to viruses that warrant further investigation.

## Non-Viral Gene Delivery to Bone

Bone tissue has the ability to repair and regenerate itself. Nonetheless, this capacity may be reduced or completely lost depending on the size of the defect (aka. critical size defect) or by the presence of specific disease states. They were going further from a healthy state of bone tissue results in clinical cases with an increase in morbidity and mortality (Vajgel et al., 2014). In this context, bone grafts are widely applied in a wide array of clinical settings to augment or induce bone regeneration and repair. Therapies currently used, such as allografts and autografts, involve numerous practical and clinical problems.

On the one hand, allografts have enhanced osteoinductivity and are relatively abundant in supply; nevertheless, they involve the potential risk to transmit disease. On the other hand, autografts are still considered as the “gold standard” for bone regeneration, as they can provide all the needed osteogenic components for bone repair. However, pain and morbidity at the donor site, a limited amount of available tissue, but also prolonged surgery are the main problems now facing this clinical approach. Nowadays, besides bone autografts and allografts, regenerative procedures are more focused on bone tissue engineering as an alternative using ceramics, polymers, and growth factors (Dimitriou et al., 2011). In combination with those scaffolds and biomaterials, factors inducing osteogenesis have been used to accelerate bone healing (Pereira et al., 2020). Many teams designed excellent delivery systems for growth factors; however, recombinant growth factors are expensive and onerous to produce (De Witte et al., 2018). Moreover, in an *in vivo* setting, high doses must be injected/administered to address the issues related to the brief half-life of the growth factors (Balmayor and van Griensven, 2015). In summary, we can say that protein delivery systems are still paved with many challenges, while gene therapy may provide a more suitable alternative.

Non-viral gene delivery/transfer is often performed using pDNA These circular, small, double-stranded DNA structures are stable, can be readily produced in bacteria and customized with a variety of different promoters (Gill et al., 2009). To be transcribed by the recipient cell, the pDNA has to reach the cell’s nucleus, and several barriers have to be overcome for this to occur. First of all, body clearance (*in vivo*) and degradation must be limited. Secondly, to be efficient, the pDNA has to cross both cell and nuclear membranes to enter the nucleus. Thirdly, the pDNA has to be released from any possible transfection complexes (Dang and Leong, 2006; Smith, 2008). To be efficient, a non-viral gene delivery is dependent on; (I) the DNA sequence, (II) preparation of the construct, (III) purification from bacterial expansion, (IV) the chosen transfection method, (V) the recipient cell type, and (VI) the cell cycle phase the recipient cells are in Table 3.

**TABLE 3:** Summary of non-viral gene delivery vector applied to bone tissue engineering.

Chemical vector	Scaffold/matrice or add-on	Wound type	Animal; Cells	Growth Factor or else	DNA/RNA	Results	References
***Lipid-based transfection/Lipid-based gene vectors***							

FuGENE 6	N.D.	*in vitro*	Fetal Rat Osteoblasts	TGF-β1	pDNA	Higher cell proliferation compared recombinant TGF-β1 delivery in the medium.	Macdonald et al., 2007
Lipofectamine 2000	N.D.	*in vitro*	BMSCs	antimiR-138	Oligonucleotide	Massive bone regeneration and with good vascularisation were achieved.	Yan et al., 2014
(DOTAP)-2-dioleoyl-sn-glycero-3-phosphatidylethanolamine	Transferrin	*in vitro*	MG63 and MC3T3-E1 cells	β-galactosidase	pDNA	High correlation between lipid formulation and transfection activity.	Oliveira et al., 2009
Cationic liposome-based reagent	N.D.	*in vitro*	Human BMSCs	GFP	pDNA	High viabilities and recoveries of the transfected cells as well as multipotency.	Madeira et al., 2010
Cationic liposome	N.D.	*in vitro*	AH130 cells	N.D.	pDNA	Efficient transgene expression as well as enhanced nuclear delivery.	Tachibana et al., 2002
DODAP, HSPC, Chol, and DSPE-PEG	Polycaprolactone (PCL) scaffolds	*in vitro*	Human BMSCs	Runx2	pDNA	Osteogenic differentiation was achieved with long-term gene expression of RUNX2.	Monteiro et al., 2014
FuGENE 6	Type-I collagen and poly(lactide-co-glycolide) (PLG) scaffolds	*in vitro*	BHK cells	N.D.	pDNA	Improvement of the functional stability and release duration.	Winn et al., 2005
Lipofectamine	N.D.	*in vitro*	Human BMSCs	BMP-2 and VEGF165	pDNA	Differentiation abilities of BMSCs were enhanced.	Guo-ping et al., 2010
Amaxa Nucleofector- II	N.D.	*in vitro*	Human primary calvarial suture MSCs	BMP-2 and BMP-3	pDNA	Efficient, a non-viral alternative method for in vitro applications.	Dwivedi et al., 2012

***Synthetic polymer-based transfections/Synthetic polymer-based gene vectors***							

Polyethylenimine (PEI)	N.D.	Intracerebral transfer	primary rat brain endothelial cells or chicken embryonic neurons.	Luciferase	pDNA	Results comparable or even better than lipopolyamines.	Boussif et al., 1995
Polyethylenimine (PEI)	N.D.	N.D.	COS-7 cells	Luciferase	pDNA	Transfection activity of PEI vectors is due to their unique ability to avoid acidic lysosomes.	Akinc et al., 2005
Polyethylenimines (PEIs) with F25-LMW Liposome	N.D.	N.D.	SKOV-3 cells	N.D.	pDNA and siRNA	Lipopolyplexes show improved biological properties over PEI complexes	Schafer et al., 2010
Polyethylenimine (PEI)-7K-L	N.D.	N.D.	293T cells	Luciferase	pDNA	PEI-7K-L is less cytotoxic and more efficient than both PEI-25K and Lipofectamine 2000 in the in vitro gene transfection	Deng et al., 2009
Polyethylenimine (PEI)	N.D.	N.D.	HeLa cells	N.D.	pDNA	PEI cannot induce changes in lysosomal pH.	Benjaminsen et al., 2013
Polyethylenimine (PEI)	N.D.	Adult (eight weeks old) OFl female or male mice central nervous system/neural disorder	Neuronal cultures	Luciferase and bcl2	pDNA	PEI appears to have potential for fundamental research and genetic therapy of the brain.	Abdallah et al., 1996
Polyethylenimine (PEI)	N.D.	N.D.	Dendritic cells	GM-CSF	pDNA	Results open new approches for novel delivery vectors for in situ vaccination and the treatment of autoimmunity.	Ali and Mooney, 2008
Polyethylenimine (PEI)	Porous poly(lactide-co-glycolide) (PLG) scaffolds	Subcutaneous implantation	Rat	β-galactosidase	pDNA	In vivo long-term and high level of gene expression.	Huang et al., 2005a
Polyethylenimine (PEI)	Poly(lactic-co-glycolic acid) (PLGA) scaffolds	Calvarial defects	Rat	BMP-4	pDNA	PEI scaffold delivery system was able to enhance bone formation.	Huang et al., 2005b
Polyethylenimine (PEI)	Collagen, collagen GAG, and collagen nHa scaffolds	N.D.	Rat MSCs	Luciferase	pDNA	PEI is a highly efficient pDNA transfection agent for both MSC monolayer cultures and 3D environment.	Tierney et al., 2012
Polyethylenimine (PEI)	Collagen scaffolds	Calvarial defects	Rat; Human BMSCs	PDGF-B	pDNA	PDGF-B gene-activated scaffolds are useful for bone regeneration.	Elangovan et al., 2014
Polyethylenimine (PEI)	Poly-(ε-caprolactone) scaffolds	N.D.	C2C12 cells	BMP-2	pDNA	PEI, as bioactive implant surfaces give rise to promising results.	Reckhenrich et al., 2012
Poly(ethyleneglycol) (PEG)	N.D.	Calvarial defects	Mice; Mouse calvarial cells	caALK6 and Runx2	pDNA	First, in vivo gene transfer with therapeutic potential using polyplex nanomicelles.	Itaka et al., 2007
Poly(ethyleneglycol) (PEG)	Poly(ethylene glycol) (PEG) hydrogels	N.D.	HEK293 cells and Human MSCs	GFP and Luciferase	siRNA	Delivery of siRNA and miRNA from the hydrogel constructs enhanced the osteogenic differentiation.	Nguyen et al., 2014

**Natural polymer-based transfection/Natural polymer-based gene vectors**							

Chitosan functionalized with imidazole moieties	N.D.	N.D.	293T and HepG2 cells	β-galactosidase	pDNA	Enhanced β-gal expression.	Moreira et al., 2009
Calcium phosphate	Chitosan	Subcutaneous implantation	Mice; MC3T3-E1 cells	BMP-2	pDNA	Bone tissue formation in vivo after implantation.	Krebs et al., 2010
Alginate hydrogel	N.D.	?	Mice; Human MSCs and MG-63 cells	BMP-2	pDNA	Alginate hydrogel seems to be highly suitable for the delivery of growth factors in bone regeneration.	Wegman et al., 2011
Alginate hydrogel	Ceramic granules	Spinal cassettes	Goat MSCs	BMP-2	pDNA	Alginate hydrogel led to stable expression of BMP-2 and promoted osteogenic differentiation.	Wegman et al., 2014
Chitosan	N.D.	N.D.	Human MSCs, MG63, and HEK293 cells	β-galactosidase	pDNA	Chitosan-DNA nanoparticles are cell type-dependent and not cytotoxic.	Corsi et al., 2003
Chitosan-alginate	N.D.	Subcutaneous implantation	Mice; HEK 293 cells and Human MSCs	BMP-7	pDNA	The chitosan-alginate gel used a gene delivery system seems to be an exciting approach for tissue engineering.	Park et al., 2007
Composites of cationized gelatin microspheres (CGMS)	Oligo(poly(ethylene glycol)fumarate) (OPF)	Subcutaneous implantation	Mice	BMP-2	pDNA	Composites can prolong and control the release of pDNA.	Kasper et al., 2005
Composites of cationized gelatin microspheres (CGMS)	Oligo(poly(ethylene glycol)fumarate) (OPF)	Calvarial defects	Rat	BMP-2	pDNA	The release of plasmid DNA from the composites was not sufficient to induce bone repair.	Kasper et al., 2006
Branched triacrylate/amine polycationic polymer with gelatin microparticles	Oligo(poly(ethylene glycol)fumarate) (OPF)	Calvarial defects	Rat; CRL 1764 cells	BMP-2	pDNA	Polycationic polymers with a slow degradation rate can prolong the release of pDNA.	Chew et al., 2011
Alginate hydrogel	Hyaluronic Acid (HA)-based Gel	Tibial defects	Rabbit	TGF-β1 and FGF-2	proteins	By angiogenesis inhibition and hypoxic environment promotion, cartilage formation can be exclusively promoted.	Stevens et al., 2005

***Inorganic nanoparticles transfection/Inorganic nanoparticles gene vectors***							

Calcium phosphate nanoparticles	N.D.	N.D.	HeLa and MC3T3-E1 cells	Luciferase	pDNA	Transfection efficiencies due to efficient condensation and bound of pDNA.	Olton et al., 2007
Calcium phosphate nanoparticles	Polyelectrolyte multilayer poly-(L-lysine) (PLL)	N.D.	Human osteoblasts	Spp1 for the silencing of osteopontin expression and Bglap-rs1 for silencing of osteocalcin expression	shRNA	A multilayered films-based delivery system containing nanoparticles for gene silencing can specific for bone cells.	Zhang et al., 2010
Hydroxyapatite nanoparticles	Collagen scaffolds	Calvarial defects	Rat; MSCs, HUVECs, MC3T3-E1s	BMP-2 and VEGF-165	pDNA	Bone regeneration was accelerated.	Curtin et al., 2015
Alginate	Ceramic granules	Spinal cassettes	Goat; Goat MSCs	BMP-2 and VEGF-165	pDNA	Transfection from this DNA delivery system led to a stable expression of BMP-2 during 16 weeks.	Wegman et al., 2014
Polyethylenimine (PEI)-LA	Gelatin/collagen scaffolds	Subcutaneous implantation	Rat	bFGF and BMP-2	pDNA	Scaffolds delivering complexes influenced recombinant protein production.	Rose et al., 2012
Lipofectamine 2000 (coprecipitated within apatite)	PLGA films	N.D.	C3H10T1/2 cells	β-galactosidase	pDNA	The hybrid material system integrates conductivity provided by the apatite and inductivity supplied by the DNA.	Luong et al., 2009

***Physical transfection methods/Physical gene vectors methods***							

Electroporation	HA/β-TCP scaffolds	Calvarial and long-bone segmental defects	Rat; ASCs	BMP-2 to VEGF-165	pDNA	Induction of rapid angiogenesis and osteogenesis.	Lee et al., 2019
TransIT-2020	Matrigel	Calvarial defects	Rat; Rat BMSCs	BMP-2	pDNA	BMSCs transfected with BMP-2 provided better osteogenic differentiation than primary BMSCs.	Hsieh et al., 2018
Sonoporation	N.D.	Ectopic implantation - Mice; Orthotropic implantation – Rat	Mice and Rat	BMP-2 and BMP-7	pDNA	Sonoporation increased callus formation and heterotopic ossification.	Feichtinger et al., 2014

***Ex vivo transfections/Ex vivo gene vectors***							

Nucleofector^TM^	Fibrin gel	Coccygeal vertebrae	Rat; Porcine ASCs	BMP-6	pDNA	ASCs modified with BMP-6 can repair vertebral bone defects.	Sheyn et al., 2011
Nucleofector^TM^	N.D.	Spinal fusion in lumbar paravertebral muscle	Mice; Porcine ASCs	BMP-6	pDNA	Formation of a large bone mass adjacent to the lumbar area, which produced posterior spinal fusion.	Sheyn et al., 2008b
Microporation transfection	Poly(lactic-co-glycolic acid) (PLGA) scaffolds	Dorsal subcutaneous spaces	Mice; Human ASCs	BMP-2 and Runx2	pDNA	The co-transfection of two osteogenic lineage-determining genes could enhance osteogenic differentiation of ASCs.	Lee et al., 2010
Lipofectamine 2000	N.D.	Osteodistraction of the mandible	Rabbit; Rabbit BMSCs	Osterix	pDNA	Promotion of bone formation.	Lai et al., 2011

***Peptides***							

protease-degradable (PEG) functionalized with a peptide (GFOGER)	N.D.	Radius defects	Mice; Human MSCs	BMP-2	protein	GFOGER hydrogels promote bone regeneration with low delivered BMP-2 doses.	Shekaran et al., 2014
(K)16GRGDSPC	Bioactive bone matricesPLGA-[ASP-PEG]n	Segmental bone defects in femoral shafts	Rabbit; Human BMSCs	TGF-β1	pDNA	The biomimetic bone matrix is a very promising scaffold to increase of bone repair.	Pan et al., 2014

***Hybrid for transfections/Hybrid as gene vectors***							

Polyethylenimine (PEI)-LA	Gelatine and collagen scaffolds	Subcutaneous implantation	Rat; 293T cells	bFGF and BMP-2	pDNA	PEI-LA was effective in vivo gene delivery carrier.	Rose et al., 2012
Organic/inorganic hybrid co-precipitated within apatite	PLGA films	N.D.	C3H10T1/2 cells	β-galactosidase	pDNA	This hybrid material system integrates inductivity provided by the DNA and conductivity provided by the apatite.	Luong et al., 2009
Cationized gelatin microspheres and OPF	N.D.	N.D.	N.D.	N.D.	pDNA	In vivo prolongation of the availability of pDNA.	Kasper et al., 2005
Cationized gelatin microspheres within a crosslinked OPF	N.D.	Calvarial defects	Rat	BMP-2	pDNA	The release of plasmid DNA from the composites was not sufficient to elicit a bone regeneration response.	Kasper et al., 2006
TAPP complexed with gelatine microparticles	poly(propylene fumarate) scaffolds	Calvarial defects	Rat	N.D.	pDNA	Slow degradation rate can prolong the release of pDNA from the composite scaffolds.	Chew et al., 2011
Chitosan-disulfide-conjugated low molecular weight PEI	N.D.	N.D.	MG-63 cells and stem cells	BMP-2	pDNA	Transfection efficiency was significantly higher than PEI and comparable to Lipofectamine.	Zhao et al., 2013

***Others***							

Electrospinning	Non-woven, nano-fibered, PLGA, PLA-PEG	N.D.	MC3T3-E1 cells	β-galactosidase	pDNA	Incorporation of pDNA into a polymer scaffold can be achieved using electrospinning.	Luu et al., 2003
Polymer Matrices	Porous poly(lactide-co-glycolide) (PLG) scaffolds	Subcutaneous implantation	Rat; 293T cells	PDGF	pDNA	Enhanced matrix deposition and blood vessel formation.	Shea et al., 1999
Gene activated matrices	Collagen I scaffolds	Femoral and tibial metaphysis defects	Dog	PTH	pDNA	Induction new bone formation.	Bonadio et al., 1999

*N.D., non determined.*

To deliver biologics to the bone fracture site to repair bone defects, gene therapy using gene vectors offers an attractive alternative method. At the delivery site, the target genes induce the production of potent growth factors (e.g., endogenous BMPs, VEGF) (Curtin et al., 2015), which is more efficient than exogenous delivery of recombinant proteins. Additionally, gene therapy induces *in situ* osteoblast differentiation, enhances osteoinduction via the expression of growth factors, and facilitates mineralized matrix production (Luo et al., 2005). Recently, non-viral gene delivery vectors, including lipids, peptides, dendrimers, and cationic polymers have been proposed as alternative strategies for gene delivery. This renewed interest is mainly attributed to their many advantages, such as the absence of endogenous virus recombination, their low immunogenicity, and tunable construction and easy fabrication (Pack et al., 2005; Mintzer and Simanek, 2009; Guo and Huang, 2012). Futhermore, many of these non-viral gene vectors have been used in clinical trials, combined with or without biomaterials (Li et al., 2016). In the following section, we summarize the most commonly used non-viral gene vectors and highlight their potential applications (Lechardeur and Lukacs, 2002; Ramamoorth and Narvekar, 2015) or more advanced ones (Pack et al., 2005; Mintzer and Simanek, 2009; Guo and Huang, 2012).

### Lipid-Based Gene Vectors for Bone

The most commonly used lipid-based delivery systems, e.g., FuGENE^TM^ and Lipofactamine 2000^TM^, have been widely used in research for several years due to their high and stable transfection efficiencies and commercially availability. On one hand, we can notably cite FuGENE6, which was used to transfer the gene TGF-β1, an osteoinductive growth factor into osteoblasts (Macdonald et al., 2007). After transfection, the osteoblasts demonstrated superior cell proliferation in comparison to cells treated with equivalent levels of recombinant TGF-β1 added to the culture medium. These results highlighted the advantages and efficiency of gene delivery instead of exogenous delivery of growth factors for bone tissue engineering (Macdonald et al., 2007). Lipofectamine 2000-based formulations have been used to deliver the oligonucleotide antimiR-138 to bone-marrow derived stromal cells (BMSCs) to form stem cell “patches.” When these sheets are applied to freeze-dried allograft bone, this induces massive bone regeneration with good vascularisation (Yan et al., 2014). Another example of lipid-based non-viral gene delivery system are the two molecules 1,2-dioleoyl-3-trimethylammonium propane (DOTAP)-2-dioleoyl-sn-glycerol-3-phosphatidylethanolamine and DOTAP-cholesterol. These two were used to deliver β-galactosidase plasmid to human and mouse osteoblastic cell lines (MG63 and MC3T3-E1, respectively). To increase the expression and efficiency of this delivery system, transferrin was incorporated into the system. The results demonstrated that this method had a higher efficiency in osteoblastic cell lines than in a human melanoma cell line (aka. 294T cell line). It also revealed a high correlation between lipid formulation, transfection activity, DNA dose, and charge ratios of the complexes (Oliveira et al., 2009; Yan et al., 2014).

Lipid-mediated gene transfer was one of the earliest strategies applied in gene therapy (Dwivedi et al., 2012), and positively charged liposomes were the first non-viral delivery vectors used in clinical trials (Li et al., 2015). Most of the time, to initiate bone progenitor cell differentiation and newly formed bone ossification, strategies have been focused on the delivery of genes encoding TGF-β and BMPs (Winn et al., 2005; Guo-ping et al., 2010). Another approach can be to target directly the master gene of bone differentiation (aka. runt-related transcription factor 2, RUNX2) with DNA plasmid encoding transcription factor RUNX2 loaded into liposomes and covalently immobilized onto polycaprolactone (PCL) nanofibers (Monteiro et al., 2014). Using BMSCs results showed that cells cultured with this setup showed a higher total protein synthesis and enhanced levels of metabolic activity (Table 3). However, even though liposome-based gene delivery was one of the first methods used to introduce exogenous DNA into eukaryotic cells, this method is not widespread in other fields like bone tissue engineering. This is possibly due to the involvement of cationic liposomes (lipoplexes), which are cytotoxic at higher concentrations (Tachibana et al., 2002; Madeira et al., 2010). For this reason, liposomes associated with scaffolds as a combined system should be used to deliver genes in a cell-controlled and spatially localized manner, for efficient bone tissue engineering applications.

### Synthetic Polymer-Based Gene Vectors for Bone

Synthetic polymers can be also used as non-viral gene carriers as they can be endocytosed by cells. A variety of molecules that can differ in chemical composition, 3D architecture, weight, side-chain length, size, and branching, or even density, are available (Park et al., 2006). Most polymers described in the literature for gene therapy are cationic (aka. with a positive charge) with mainly amines groups (Santos et al., 2011). These positive groups interact with the negatively charged phosphate groups present in the DNA sequence and after association form condensed structures called polyplexes.

PEI, one of the first and most successful polyplexes used as non-viral gene vectors (Pack et al., 2005), was first introduced in 1995 both *in vitro* and *in vivo* (Boussif et al., 1995). PEI as a non-viral vector has several critical advantages over viral vectors; (I) it is less cytotoxic, (II) less immunogenic, (III) there are no carcinogenic concerns, (IV) it induces transient gene expression, and (V) it is safe for clinical use (Pack et al., 2005). Additionally, PEI has a high transfer efficiency (Akinc et al., 2005; Deng et al., 2009; Schafer et al., 2010) due to a phenomenon known as “proton sponge effect” (Benjaminsen et al., 2013). The transfection efficiencies are comparable with viral gene delivery agents (Abdallah et al., 1996). Numerous publications have highlighted the branched 25 kDa PEI polymer as the most widely utilized gene transfer agent (Huang et al., 2005a,b; Ali and Mooney, 2008) and as a “gold standard” (aka. positive control) across *in vitro* studies (Park, 2009). In brief, PEI combined with pDNA as polyplex have properties that can be changed by merely altering the PEI amines/DNA phosphates ratio. Higher ratios of PEI to pDNA usually result in higher transfection efficiencies, but the downside is an increased cytotoxicity (Boussif et al., 1995; Godbey et al., 1999). To optimize the use of polyplexes for gene transfer for bone tissue engineering applications, a balance between efficiency and cytotoxicity must be reached (Tierney et al., 2012).

To achieve the above, collagen scaffolds can be used to incorporate the complex branched PEI (25 kDa) with pDNA (Elangovan et al., 2014). The use of gene-activated scaffolds (with pPDGF-β) in a calvarial defect rat model, favored cell attachment and promoted cell proliferation *in vitro*. It was also described to promote osteogenesis (osteoinduction and osteoconduction) and demonstrated superior tissue regeneration when compared to empty scaffold and empty calvarial defect groups. Another documented polyplex is the combination of PEI (branched, 25 kDa)/pBMP-2, in association with a poly(ε-caprolactone) scaffold. This combination was applied to initiate *in vitro* differentiation of myoblasts (Reckhenrich et al., 2012). With optimized gene doses, cells increased the secretion of osteocalcin and osteopontin compared to the control group, demonstrating transdifferentiation of C2C12 cells into the osteoblastic lineage.

As a last example of polyplex, we can cite the advanced system consisting of dural plasmids, polyethyleneglycol (PEG)-block-catiomer (PEG-b-P[Asp-(DET)]) and a CaP-cement scaffold. This system has a high bio-compatibility rate with plasmids encoding osteogenic factors, activin receptor-like kinase 6 (caALK6) together with RUNX2 (Itaka et al., 2007). With this delivery system, osteogenic differentiation was enhanced compared to PEI or FuGENE6 (Itaka et al., 2007). Another study used branched PEI (25 KDa) with siRNA or miRNA to create complexes encapsulated within the PEG hydrogel, to deliver nucleic acids directly *in situ*. The goals of this study were to guide stem cells through osteogenic lineage with localized and sustained RNA release (Nguyen et al., 2014).

### Natural Polymer-Based Gene Vectors for Bone

Natural polymers have been used due to their lower cytotoxicity and enhanced biocompatibility compared to synthetic polymers. Chitosan is one of the most studied natural polymers in bone tissue engineering (Raftery et al., 2013). Biodegradable and biocompatible, chitosan is formed by deacetylating chitin and can be used as a gel or as micro/nanoparticles (Moreira et al., 2009; Garcia-Fuentes and Alonso, 2012) to form complexes with pDNA. Compared with liposomes, the transfection efficiency of chitosan is always a little bit lower (comparable to naked DNA), but it is significantly less toxic than liposomes and easy to work with (Corsi et al., 2003). To overcome the problem of lower transfection efficiencies, chitosan is combined with other biomaterials. For orthopedic applications, it can be incorporated into titanium films with pDNA for BMP-2 or even incorporated in alginate hydrogel as nanoparticles (Park et al., 2007). In addition to chitosan, alginate has also been utlized for gene delivery. It has many advantages such as; (I) it is non-toxic, (II) bacteriostatic, (III) anti-inflammatory, (IV) biocompatible, and (V) form of nanoparticles or be combined with other hydrogels (Krebs et al., 2010). The use of alginate-mediated transfections with pDNA was characterized by high transfection efficiency, slow release kinetics, *in vitro* osteogenic differentiation, and *in vivo* bone formation (Wegman et al., 2011, 2014). It has been applied in bone tissue-engineering applications both *in vitro* and *in vivo* (Bourgeat-Lami, 2002; Stevens et al., 2005). Gelatin as another well-known natural polymer that has been widely used in bone tissue engineering as a delivery system for DNA and growth factors (Kasper et al., 2005; Kasper et al., 2006; Chew et al., 2011). In general, natural polymers, are often easy to work with, are readily available and rarely trigger immune responses. Yet they are not widely utilized gene-delivery systems for tissue engineering. Apart from polyplexes or lipoplexes, these natural polymers are also often combined with other materials such as ceramics or synthetic polymers to be closer to biomechanical, osteoconductive, and osteoinductive properties of the targeted tissue.

### Inorganic Nanoparticles Gene Vectors for Bone

New studies have demonstrated the use of inorganic nanoparticles as a NVGD method (Bourgeat-Lami, 2002; Chowdhury and Akaike, 2005). These methods consist mostly of coupling small material particles such as iron oxide, silica, gold, or even calcium phosphate (CaP) with plasmid DNA. These particles deliver the pDNA into the cell via endocytosis. CaPs particles are favored in bone regeneration for their capacity to increase the strength and stiffness of the constructs. CaPs possess numerous advantages, which include; (I) excellent stability, (II) are biodegradable and biocompatible, (III) good solubility, (IV) good resorbability, (V) good binding affinity to DNA, and (V) efficient cellular uptake (Olton et al., 2007). CaPs present lower toxicity than carbon nanotubes, silica, magnetic particles, or quantum dots (Olton et al., 2007). CaP nanoparticles have also been combined with shRNA (Olton et al., 2007). When applied to human osteoblasts, this system showed efficient bone formation (Olton et al., 2007). Related to CaPs and known as the mineral component of bone Hydroxyapatite (HA) can also be used as a component of the NVGD strategy (Uskokovic and Uskokovic, 2011). Another related example, the nanohydroxyapatite (nHA) vector can deliver pDNA encoding for VEGF and BMP-2 to MSCs, and as a result, can markedly enhance bone healing and tissue vascularisation (Curtin et al., 2015). While these methods demonstrate some limitations such as moderate transfection efficiency and retention within the circulation, they do show several advantages, such as; (I) easy storage ability, (II) low toxicity, and (III) reasonable shape control. As a consequence, more and more studies are utilizing inorganic nanoparticles (Parveen et al., 2012).

### Physical Gene Vector Methods for Bone

Physical transfection methods involve permeabilization of the cell membrane, allowing pDNA to enter the cells. Different methods are used to permeabilize the cell membrane “in a safe way,” such as electroporation, which uses a high-intensity electric pulse. This method is not very often used but can present interesting results in the context of bone tissue engineering (Lee et al., 2019). With a transfection efficiency reaching 70–75%, BMP2 gene transduction using electroporation for the functional enhancement has been shown to enhance the *in vivo* osteogenic potential of human bone-marrow-derived mesenchymal stromal cells (hBMSCs) and adipo-tissue-derived stromal cells (ASCs), alone, or in combination with other factors (Hsieh et al., 2018).

Sonoporation disrupts the cell membrane using ultrasound to induce transfection. However, this method is not very successful and is considered as a highly experimental procedure since cell death is high. To compensate for the lower efficacy of this NVGD method, a highly osteoinductive co-expression strategy was investigated using BMP 2 and BMP-7 with significant results (Feichtinger et al., 2014) (Table 3). When sonoporation was directly compared with passive gene delivery, it demonstrated an increased probability of gene expression and bone formation related to the ultrasound energy applied. However, bone-related gene expression levels and bone volumes were not increased.

All physical methods, however, destabilize the cell membrane temporarily, which in many cases leads to low cell survival. The problem of those techniques is to search out the optimal conditions. One more difficulty is to reach deep into the tissue. These techniques are mainly capable of penetrating the skin and might maybe reach the adipose tissue and muscle just under the skin. However, bone cannot be reached with non-invasive methods, making it less optimal for orthopedic applications.

### *Ex vivo* Gene Vectors for Bone

For all the non-viral gene therapy technics/approaches described above and applied in bone tissue engineering for bone regeneration, many hurdles need to be overcome as most of the techniques are based on particle uptake and controlled cell membrane damage. After described the techniques above, we can say that the main disadvantages of *in vivo* application are; (I) low penetration depth, (II) high levels of cell death and tissue damage, (III) chances of off-target effects, and (IV) risk of particle migration. Doing *ex vivo* transfections could be one way to overcome those issues. In that case, the DNA is not directly transferred into the body to the cells of interest; however, in a multiple steps protocol, the desired host cells are (I) isolated from the body, (II) transfected *in vitro* followed by a selection, and (III) and “grafts” back to the host to act as protein factories or directly as bone-forming cells. The two main advantages compared to *in vivo* transfections are the step pre-selection of the cells of interest and the post-selection of the transfected cells. This step of quality control of the used cells increases the safety of this NVGD strategy. As safety is one of the main concerns in bone regenerative medicine, the *ex vivo* NVGD model seems to be more potent at the moment in the context of clinical applications (Sheyn et al., 2008b, 2011; Lai et al., 2011). However, the harvesting of autologous cells arises with a disadvantage, with additional surgery and time-spending (Aggarwal et al., 2010).

### Peptides as Gene Vectors for Bone

Peptides, as the NVGD method, are generally used to enhance membrane activity and targeting ability. We can notably cite as an example, a paper where a system using PEG synthetic hydrogel, functionalized with a collagen-mimetic peptide (aka. GFOGER) (Shekaran et al., 2014). In this study, the hydrogel was applied to murine bone critical-sized defects, and the authors demonstrated that this functionalized hydrogel provided increased osteoprogenitor localization in the defect site, sustained *in vivo* release of encapsulated molecules, enhanced bone formation, and induced defect bridging. With respect to these results, this system demonstrated great potential for gene delivery despite being developed initially for BMP-2 delivery. In another study, TGF-β1 was delivered by a novel NVGD vector called (K)16GRGDSPC chemically linked to a bone scaffold made with PLGA. Applying this TGF-β1 functionalized scaffold to rabbit critical size bone defects significantly increased bone regeneration compared to control groups (Pan et al., 2014).

### Hybrids as Gene Vectors for Bone

To combine many of the beneficial effects of NVGD methods, hybrid delivery systems can be an attractive approach, in particular, lipid and polymer integrated materials. PEI modified with linoleic acid and combined with different scaffolds such as collagen and gelatine as vehicles was used to study the expression levels of FGF-2 and BMP-2 after implantation in rat subcutaneous pockets (Rose et al., 2012). Another example, consisting of an organic/inorganic hybrid of pDNA-Lipoplex complex co-precipitated within apatite and loaded onto PLGA sheets, was investigated to integrate both osteoconductivity and osteoinductivity (Luong et al., 2009). Results demonstrated that the organic/inorganic hybrid resulted in improved transfection efficiency in all groups. To conclude, the co-precipitation of the DNA-lipoplexes within apatite also resulted in higher stability and better spatial distribution of DNA delivery (Luong et al., 2009).

Another option could be to combine natural and synthesized polymers to optimize NVGD systems. A NVGD consisting of a positively charged gene vector within gelatine microspheres and combined with a hydrogel of a crosslinked oligo (PEG-fumarate) (OPF) was used to investigate the effects of pBMP-2 in a critical-size rat cranial defect model on bone formation (Kasper et al., 2005). Surprisingly, there was a lack of improvement in bone regeneration, possibly due to an insufficient release of the DNA from the hydrogel (Kasper et al., 2006). Another team investigated the delivery of pBMP-2 using a biodegradable branched triacrylate/amine polycationic polymer (TAPP) that was combined with gelatine microparticles loaded within a porous tissue-engineered scaffold. In this study, they investigated the interplay between gelatine degradation, TAPP degradation, pDNA release, and mineralized matrix production in a rat calvarial critical-size defect model. The data showed that the hybrid composite scaffolds did not generate an enhanced bone regeneration in a critical-size rat cranial defect, as analyzed by microcomputed tomography and histology. These results claim, however, those polycationic polymers with a slow degradation rate can prolong the release of pDNA from composite scaffolds and suggest that gelatin microparticles comprising biodegradable polycationic polymers could be established to release pDNA in an intact polyplex form (Chew et al., 2011).

New approaches were emerging recently using the engineered matrices as a vector for targeted DNA construct, most of the time in the form of a plasmid. Multiple studies have shown that *in vivo* implantation of gene activated scaffolds/hydrogels/matrices/complexes at sites of bone defect was linked with expression of pDNA and retention for at least 6 weeks. This was followed by the induction of newly formed bone in a reproducible, stable, time-dependent, and dose-dependent manner (Bonadio et al., 1999).

## Discussion and Conclusion

NVGD methods stay in the focus of current research because of promising results in various areas of orthopedic research. We have shown that the clinical trials registered until now are mainly based in the area of bone, followed by hip, shoulder, and tendon for musculoskeletal diseases. However, the number of publications on non-viral gene delivery is not directional proportional to the interest in clinical trials in the different joints and tissues. It seemed that most literature was found for bone repair, followed by cartilage, IVD, and ligament approaches.

A common problem of all non-viral methods seemed to find promising solutions to deliver DNA or RNA with musculoskeletal specific cells in connective tissues. Significant conceptual differences exist between gene delivery methods to isolated cells *in vitro* and to *in vivo* or *ex vivo* to tissue. The current literature demonstrates the enthusiasm and powerful approach of non-viral gene systems to the areas of bone and joint diseases. The limited number of clinical trials related to non-viral gene delivery may also reflect some of the challenges that the field of gene therapy has faced over the past decade due to safety concerns related to viral vectors. However, as this review demonstrates, many NVGD methods have significant potential but require further protocol optimization or longer-term animal studies to determine their efficacy. Indeed it appears that efficacy and efficiency of the therapeutic strategy whether it is cell proliferation or structural restoration of soft or hard tissue, remains one of the significant challenges of NVGD systems. It is likely that a “one-shoe fits all” approach will not work for all orthopedic tissues, and a more targeted approach dependent on cell type, tissue composition/structure, and disease state/defect size will be necessary.

Cytotoxicity of viral vectors and the risk of host integration of these genomes, which might cause unpredicted gene mutations of the host genome, are clear contra-indicators for viral gene therapy. Conversely, non-viral gene therapy methods are on the rise, and here a tremendous variety of delivery methods exist, as we have listed in this review. In terms of clinical translation from *in vitro* to *in vivo*, a significant hurdle is transducing an adequate number of cells to enhance the therapeutic parameters of interest in the target tissue of interest. This can be challenging for orthopedic tissues that are relatively acellular such as the IVD and cartilage but might not be as difficult for repairing bone that is more cellular and vascularized. However, transfection efficiency has to be optimized while also taking into account any effects of cytoxicty, which has been observed for NVGDs such as cationic polymers and electroporation. On the flip-side, increased cellularity and vascularization of bone could lead to off-target and even unwarranted responses in other tissues but is likely not a problem for disk or cartilage. Disease state also needs to be considered when transitioning from *in vitro* to *in vivo* and the ability of NVGD systems to transduce cells within a degenerate tissue environment that is often catabolic and inflammatory. This may result in increased turnover, degradation and clearance of the NVGD system, limiting overall efficacy of the therapeutic strategy and optimizing NVGD systems to take these parameters into account, for example, creating polyplexes within MMP-degradable hydrogels for therapeutic release (Feng et al., 2018) or EVs that can package multiple genes targeting both tissue regeneration and inflammation. Also, to direct *in vivo* translation, *ex vivo* culture offers an alternative route whereby cells can be extracted, manipulated *in vitro* with NVGD systems, and then reinserted back into the patient, similar to what is currently being done for autologous chondrocyte implantation therapy (Krill et al., 2018). This circumvents problems around transfection efficiency *in vivo*. However, it often involves harvesting cells/tissue from healthy regions and also significant expansion time *ex vivo*.

In our view, on the side of carrier-based NVGD, the future research and potential lie in the areas of EVs in the combination of miRNA or lncRNA transmission that influence the host cells with specific functions. Here, we have seen tremendous potential, with many groups that are interested in how OA or IVDD could be targeted by transient modification of BMPs and or inflammatory genes or genes of the ECM, depending on the application in orthopedics. One of the key attractive features of NVGD is safety and low immunogenicity. Lipid-based vectors can be readily endocytosed, tissue-nano-transfection offers a safe and specific method to transfect single cells with high efficiency, polymers, both natural and synthetic can be hybridized to increase the efficiency of delivery and EVs can be generated from autologous cells packaged with a number of gene vectors.

On the side of carrier-free and physical methods how to overcome the cellular membrane, we found that electroporation (nucleofection) has been applied by many studies with relatively high efficiencies, both *in vitro* and *in vivo* directly on tissue. EVs are attractive NGVD systems as they demonstrate minimal immunogenicity, can be readily generated from autologous human cells in large quantities, can be endocytosed, and loaded with gene vector of interest. Furthermore, an interesting and exciting area is the use of tissue nano-transfection, which has high clinical value with the ability to transfect single cells in vivo and, in turn generating endogenous EVs with genetic cargo (Gallego-Perez et al., 2017).

For the IVD, NVGD methods have been primarily investigated *in vitro* with some studies using organ culture or *in vivo* rat or rabbit models. The type of vectors that have been investigated range from anti-inflammatory/fibrotic agents, siRNA targeting anti-apoptotic/catabolic enzymes, or discogenic growth factors and transcription factors. For the disk, specific considerations that apply include transducing a relatively acellular tissue. These particular tissue regions may require different vectors (NP versus inner or outer AF), ECM (negatively charged proteoglycans), and disease state. Most *in vivo* studies have focused on utilizing synthetic polymers with some success and therefore highlighting these NVGD methods (Feng et al., 2018). However, emerging/future areas that could be used by the IVD could include EVs which could be readily injected or tissue nano-transfection that could be applied directly to the disk surface. Furthermore, to truly assessing the safety and efficacy of NVGD methods for treating painful IVD degeneration and regenerating the IVD, utilizing relevant animals and assessing parameters that include pain behaviors seems paramount.

## Author Contributions

BG initiated the review, performed the major literature search on PubMed, wrote the sections on cartilage repair, drafted major parts of the MS, painted and created the figures, and provided the funding. ST provided the sections on EVs. DP provided the chapters on lipofection and IVD regeneration, language in the “Discussion and Conclusion” section, provided the funding, and edited the manuscript. JG provided the section on bone regeneration. AG provided the text on the non-viral gene therapy methods and electroporation. AC approved and edited the text. NH-C and AS-P provided the sections on the advantages and disadvantages of NVGD and introduction to synthetic polymer-based gene vectors. All the authors approved the final version of the manuscript.

## Conflict of Interest

The authors declare that the research was conducted in the absence of any commercial or financial relationships that could be construed as a potential conflict of interest.

## Abbreviations

AAV: adenoviral vector
ADAMTS5: A Disintegrin and Metalloproteinase with Thrombospondin motifs 5
ASCs: adipo-tissue-derived mesenchymal stromal cells
BMPs: bone morphogenic proteins
CAP: chondrocyte-affinity peptide
CGMS: composites of cationized gelatin microspheres
GAG: glycosaminoglycans
GMP: good manufacturing practice
GDS: gene delivery systems
HA: hyaluronic acid
hBMSCs: human bone-marrow-derived mesenchymal stromal cells
hTERT: human telomerase reverse transcriptase
EP: electroporation
ECM: extracellular matrix
EVs: extracellular vesicles
FITC: fluorescein isothiocyanate
IVD: intervertebral disk
IVDD: intervertebral disk degeneration disease
LNPs: lipid nucleic acid nanoparticles
LTI: lysine-tri-isocyanate
MiRNA: micro-RNA
MMP: matrix metalloproteinase
MSC: mesenchymal stromal cell
MPMs: mixed polyplex micelles
MVs: matrix vesicles
N.D.: non-determined
NVGD: non-viral gene delivery
OA: osteoarthritis
PAMAM: polyamidoamine dendrimers
pBMP-2: plasmid containing BMP-2
PDGF: platelet-derived growth factor
pDNA: plasmid DNA
PEI: poly-ethylene-imine
PLGA: Poly(D,L-lactide-coglycolide) lactide:glycolide acid
PTH: parathyroid hormone
siRNA: silencing RNA
VEGF: vascular endothelial growth factor.
